# Human ISG15 deficiency unveils impaired healing of ulcerations via type I interferon–mediated fibrosis

**DOI:** 10.70962/jhi.20250011

**Published:** 2026-02-24

**Authors:** Christos Sazeides, Lorenzo Cuollo, Ikjot Sidhu, Haley E. Randolph, Marta Martin-Fernandez, Sofija Buta, O’ Jay Stewart, Rachel Geltman, Jonas A. Adalsteinsson, Robert G. Phelps, Amir Horowitz, David Stein, Mariam Youssef, Andres Martinez-Muniz, Michelle Hernandez, Michail S. Lionakis, Alexis Boneparth, Joshua D. Milner, Shruti Naik, Dusan Bogunovic

**Affiliations:** 1 Center for Genetic Errors of Immunity, Columbia University, New York, NY, USA; 2 Department of Pediatrics, Columbia University Medical Center, New York, NY, USA; 3 Precision Immunology Institute, Icahn School of Medicine at Mount Sinai, New York, NY, USA; 4 Department of Immunology and Immunotherapy, Icahn School of Medicine at Mount Sinai, New York, NY, USA; 5 Department of Dermatology, Icahn School of Medicine at Mount Sinai, New York, NY, USA; 6 Lipschultz Precision Immunology Institute, Icahn School of Medicine at Mount Sinai, New York, NY, USA; 7 Tisch Cancer Institute, Icahn School of Medicine at Mount Sinai, New York, NY, USA; 8 Department of Genetics and Genomic Sciences, Icahn School of Medicine at Mount Sinai, New York, NY, USA; 9 Division of Allergy, Immunology and Rheumatology, Department of Pediatrics, University of North Carolina, Chapel Hill, NC, USA; 10 National Institute of Allergy and Infectious Diseases, National Institutes of Health, Bethesda, MD, USA; 11 Rare Diseases Research Institute, Instituto de Salud Carlos III, Madrid, Spain

## Abstract

ISG15 deficiency is a type I interferonopathy characterized by elevated circulating type I interferon (IFN-I), intracranial calcifications, fibrotic skin lesions, and occasionally inflammatory lung disease. However, the mechanisms driving immune-mediated fibrosis remain poorly understood. In this study, we combined molecular biology approaches with spatial transcriptomics in ex vivo patient samples and in vitro models to characterize a novel loss-of-function ISG15 variant and to elucidate disease-associated molecular pathways. Analysis of patient skin biopsies revealed increased IFN-I–dependent apoptosis, altered macrophage polarization, enhanced epithelial-to-mesenchymal transition (EMT), and myofibroblast activation. In vitro, ISG15 knockout (KO), but not wild-type (WT), epithelial cells were predisposed to EMT following combined TGF-β and IFN-I stimulation. Additionally, ISG15 KO fibroblasts displayed impaired mechanical wound healing and increased IFN-I–induced apoptosis compared with WT cells. Collectively, our findings suggest that chronic IFN-I exposure in ISG15 deficiency disrupts wound repair, skews macrophage activation, and promotes EMT, contributing to fibrosis across multiple organ systems.

## Teaser

Human ISG15 deficiency is a monogenic syndrome that poises individuals to a proapoptotic and profibrotic state that can lead to lung and skin damage with scarring and impaired function via chronic inflammation.

## Introduction

Type I interferonopathy is an umbrella term for monogenic diseases that lead to augmented activation and response to type I interferons (IFN-Is)—potent antiviral and inflammatory cytokines (1, 2). Type I interferonopathies can be the result of high production of IFN-I due to mutations in genes required for proper nucleic acid metabolism (e.g., TREX1 [3], SAMHD1 [4], ADAR1 [5, 6, 7]), sensing [e.g., RIG-1] [8], IFIH1 [9]), and/or increased signal amplification downstream of sensing (e.g., STING [10], COPA [11]). Such mutations lead to accumulation of endogenous ligands or gain-of-function of the pathway, all resulting in subsequent increased production of IFN-I (1, 2). An alternative cause of type I interferonopathies is the enhanced responsiveness to IFN-I downstream of its cognate receptor IFNAR (encoded by *IFNAR1* and *IFNAR2*), and thus hyperactivity of JAK1, TYK2, STAT1, STAT2, and IRF9, all resulting in subsequent increased transcription of IFN-I–stimulated genes (ISGs) (12). This can be due to the gain-of-function mutations of signal transducers (e.g., JAK1 [13], STAT1 [14])-which also affect other pathways- due to loss-of-function mutations in negative regulators (e.g., USP18 [15, 16], interferon-stimulated gene 15 [ISG15] [17]) of the IFN-I pathway (1, 2, 18), or due to biallelic mutations in the very defined region of STAT2, responsible for aiding the function of USP18 (19). While type I interferonopathy patients have several canonical shared features, such as perpetual elevation of IFN-I in their blood, ensuing elevation of ISGs, and bilateral basal ganglion calcifications, a few other clinical features and likely associated pathologies can be genotype-specific (1, 20, 21, 22).

The defining features of many canonical type I interferonopathies that are IFN-I–hyperproducing, such as Aicardi–Goutières syndrome (AGS), are chilblains, purpuric papules, and nodules that occur at the end of extremities, where the local body temperature is lower. AGS patients have not been reported to present with any lung-associated pathologies. In contrast, type I interferonopathies where there is continual ISG transcription and/or IFN-I signaling, as exemplified by gain-of-function mutations in STING and loss-of-function mutations in ISG15, respectively, can present with inflammatory lung pathologies. In addition, in ISG15 deficiency, patients can present with skin lesions, but these are not chilblain-like, and are found on the back, torso, and upper thighs (23, 24, 25). Thereby while very alike, continuous IFN-I signaling mechanisms can be blocked via buildup of negative regulators, while ISG15-deficient cells continually transcribe ISGs, driving differing pathological manifestations.

ISG15 is a negative regulator of IFNAR. It exerts its negative regulation by binding to USP18 and thereby sequestering it from proteasomal degradation (17). USP18 displaces JAK1 from IFNAR2 and thus halts the signal transduction. Moreover, free secreted ISG15 can help induce IFNγ in CD8 T cells and natural killer (NK) cells via LFA-1 (24, 26). Additionally, ISG15 can be conjugated to nascent proteins in a process called ISGylation by the sequential action of ubiquitin enzymes UBE1L, followed by UBCH8 and then HERC5 (27, 28, 29, 30). While this process is well described, the functional consequences of ISGylation remain poorly understood.

Individuals with ISG15 deficiency present with symptoms and signs encompassing basal ganglion calcifications, spontaneous skin lesions (which are mostly not chilblain-like but can present with subcutaneous perivascular lymphocytic inflammation without vasculitis), and interstitial lung disease, described in one patient prior to this report (25, 31). In vitro studies have shown the importance of ISG15 in skin architecture and integrity with observed dysregulation of collagen synthesis and TGFβ responses (25). In addition, ISG15 deficiency can present with Mendelian susceptibility to mycobacterial disease at least in part due to its extracellular activity (24, 26). While we and others have defined a number of genetic causes of ISG15 deficiency and have provided biochemical details of how enhanced IFN-I signaling ensues, the pathophysiology of peripheral tissue damage still remains to be explored.

Herein, we investigate pathophysiology of ISG15 deficiency through the lens of clinically evident inflammation-driven tissue damage and report a novel disease-causing mutation in *ISG15*.

## Results

### Novel ISG15 mutation leads to complete loss of ISG15 expression

**Figure 1.**
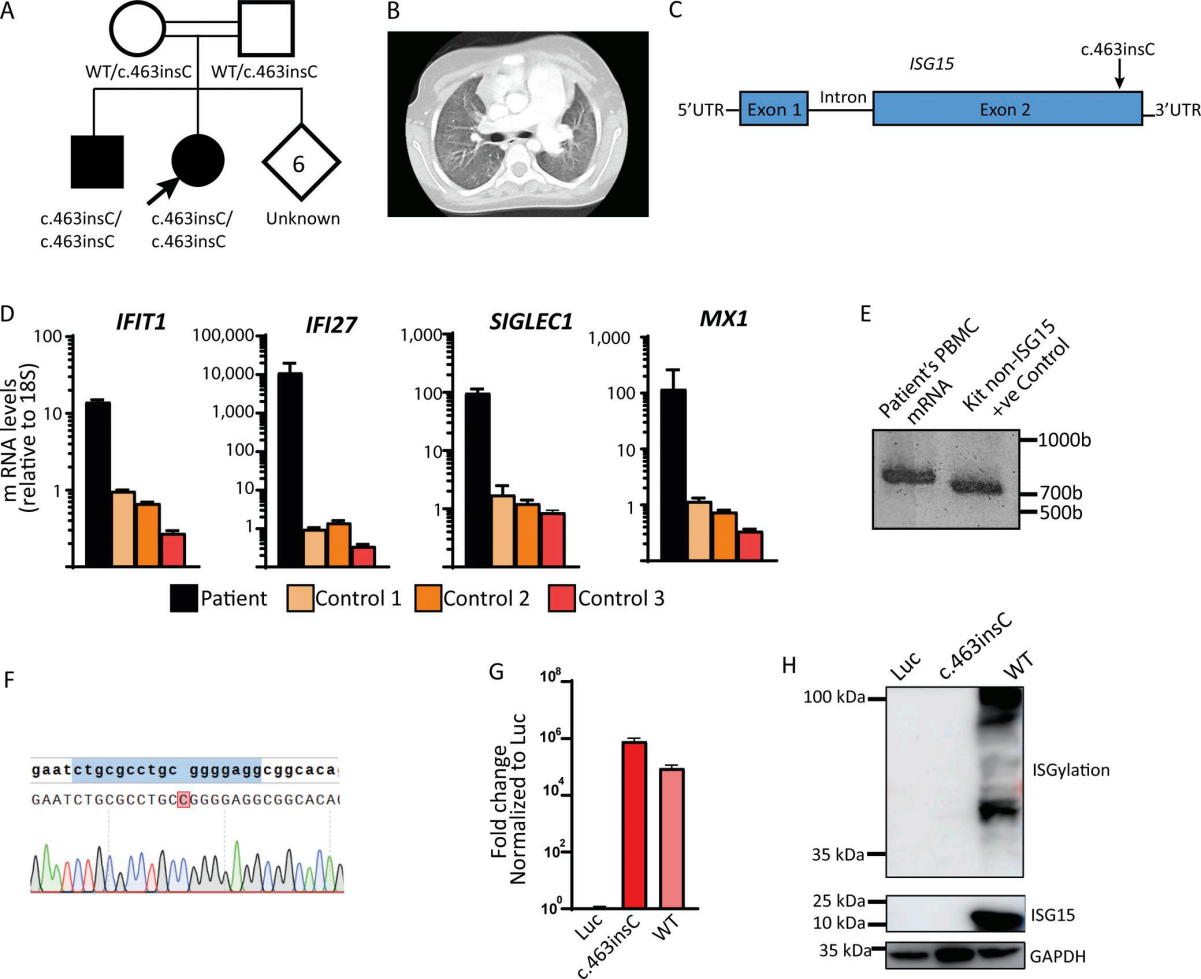
**Identification of a patient with a novel ISG15 mutation. (A)** Family includes two affected children carrying a homozygous variant (c.463insC) on *ISG15* and six children of unknown status. The arrow indicates the patient described in this section (proband). **(B)** CT of the younger sibling taken at age 12. Extensive diffuse ground-glass opacities with subpleural sparing and enlargement of the main pulmonary artery are observed. **(C)** Schematic localization of the ISG15 variant in the genomic DNA locus indicated by the arrow. **(D)** Expression levels of various ISGs (*IFI27*, *MX1*, *SIGLEC1*, and *IFIT1*) were assessed from patient’s whole blood, as well as from the three healthy controls. Target genes were normalized on 18S rRNA expression. **(E)** 3′-RACE PCR targeting *ISG15* was performed on RNA isolated from patient’s whole blood. **(F)** Sanger sequencing of the 3′-RACE band from E. **(G)** HEK293T cells were transfected with a plasmid encoding the *ISG15* variant (c.463insC), *ISG15* WT, or Luc. Relative mRNA levels for *ISG15* were assessed by qRT-PCR, performed twice for each variant, with technical triplicates; the data from one representative experiment (*n* = 3) are shown. The expression level of *ISG15* was normalized to *GAPDH*. **(H)** HEK293T cells were transfected with a plasmid encoding the ISG15 variant (c.463insC), ISG15 WT or Luc, plus HERC5, UBE1L, and UBCH8, as described in (23). Cell lysates were analyzed by western blotting for ISG15 and ISGylation; a representative experiment is shown. CT, chest tomography. Source data are available for this figure: SourceData F1.

We studied the case of a now 16-year-old female (P1) from Qatar born to consanguineous parents. The patient did not have a significant past medical history but progressively developed shortness of breath leading to her hospitalization. Pulmonary function tests showed severe restrictive lung disease whereby the lung capacity was 27% of predicted value. Additional tests revealed hypoxemia with no associated sleep-related breathing disorder and secondary pulmonary hypertension. No associated infection was detected through QuantiFERON-TB Gold (negative result) and bronchoalveolar lavage. Whole-exome sequencing revealed a homozygous mutation in *ISG15* (c.463insC) leading to frameshift and loss of the gene’s stop codon (p.Arg155Pro.fs*). The patient’s older brother (P2) (harboring the same homozygous mutation in *ISG15*) also developed severe pulmonary fibrosis that eventually led to right ventricular dysfunction and pulmonary hypertension at the age of 25. At the age of 28, he underwent double lung transplantation. Pneumonectomy revealed end-stage lung disease in the left and right lungs (an irreversible condition where lungs can no longer adequately provide oxygen to the body) with severe interstitial fibrosis, which was consistent with the clinical presentation of this patient. Moreover, pneumonectomy also showed the presence of several reactive lymph nodes in both lungs, a sign of inflammation (Fig. 1, A–C). To avoid pulmonary fibrosis and disease progression to the point of pulmonary transplant, P1 was placed on oral steroids and a JAK1/JAK2 inhibitor (ruxolitinib). Prior to treatment, we quantified the expression of four different ISGs (*IFI27*, *MX1*, *SIGLEC1*, and *IFIT1*) with quantitative RT-PCR (qRT-PCR) from patient’s whole blood. As expected, the ISG score was elevated as compared to three healthy controls (Fig. 1 D).

To assess the effect of this mutation on ISG15 expression, production, and function, we first performed ISG15-targeted 3′-rapid amplification of cDNA ends (RACE) assay to detect the presence of *ISG15* RNA, as well as of any possible isoforms. The presence of a singular band of ∼800 bases indicates that only one isoform is produced (Fig. 1 E). Sanger sequencing of this band confirmed the patient’s mutation (Fig. 1 F). Then, we evaluated mRNA expression, protein production, and function. We transfected HEK293T cells with luciferase (Luc), ISG15 variant (c.463insC), or WT ISG15. We first assessed the corresponding mRNA expression. The mutant transcript did not undergo nonsense-mediated decay as observed by the comparable levels of mRNA between the mutant and WT ISG15 (Fig. 1 G). While transcript levels were unaffected, the mutation produced a complete loss of protein, as indicated by the complete loss of detectable free ISG15 and loss of ISGylation levels (Fig. 1 H). The data above suggest the identification of a novel biallelic mutation in *ISG15* in two siblings. This mutation results in normal mRNA expression and complete loss of protein production. Given the patients’ clear clinical presentation of lung tissue damage, we sought to investigate the pathophysiology of tissue inflammation in ISG15 deficiency.

### Previous ISG15-deficient patient’s skin biopsies show areas of necrosis and high ISG signature

While lung biopsies from the patient or her brother were not available, we did have access to skin biopsies from three different lesions of a previously characterized ISG15-deficient patient (23). The patient is a female from the United States born in 2015 to nonconsanguineous parents; she had compound heterozygous variations in *ISG15* c.310G>A (p.Val104Met) and c.352C>T (p.Gln118*), leading to almost complete loss of ISG15 production. At 2 years of age, the patient developed a right inguinal cutaneous lesion characterized by a hypopigmented linear band with foci of inflammation and underlying induration. Over the following year, the lesion progressed to involve the suprapubic and lower abdominal regions, forming ulcerating plaques. Two biopsies were obtained from the lower abdominal region 20 days apart (corresponding to lesion 1 and lesion 2), and a third biopsy was obtained 2 mo later from the suprapubic region (lesion 3).

**Figure 2.**
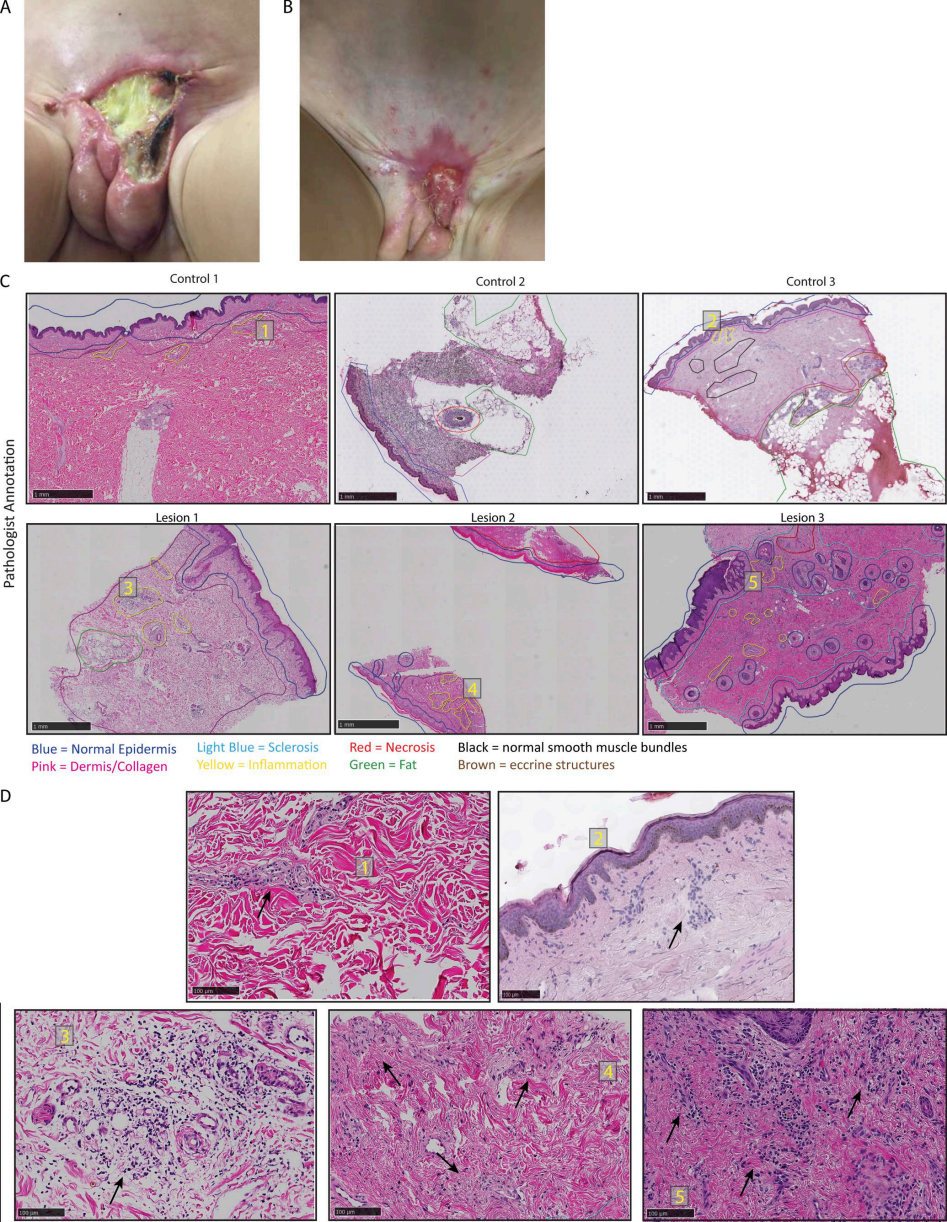
**Pathologist annotation of healthy volunteer and patient skin biopsies. (A)** Lesional skin of previously characterized patient from where three biopsies were collected at different time points. **(B)** Partial healing of lesions with extreme scarring within 2 years of first detection. **(C)** Pathologist’s annotations of three healthy volunteers and three patient’s biopsies indicating different healthy and pathological states of the tissues. **(D)** Pathologist’s annotations pointing out signs of inflammation in the skin biopsies as indicated by the black arrows. Yellow numbers in panel D indicate the magnified areas from panel C.

Histopathological analysis of lesion 1 showed subcutaneous perivascular lymphocytic inflammation without evidence of vasculitis; panniculitis with neutrophil infiltration was also observed; tissue necrosis or fungal elements were not detected. Lesion 2 on the contrary showed regions of normal and necrotic skin, with abundant *Aspergillus* hyphae infiltrating the subcutaneous tissue. Previous administration of topical corticosteroids had exacerbated the ulceration. The affected tissue was surgically resected, and the lesion eventually healed with severe scarring ∼2 years after the onset (Fig. 2, A and B) (23).

The three biopsies, along with three healthy control skin biopsies, were stained with hematoxylin and eosin (H&E) and annotated by a dermatopathologist (Fig. 2, C and D). We identified normal collagen (pink outline), normal epidermis (blue outline), and a small number of inflammatory cells, primarily lymphocytes, in healthy control and more prevalent infiltration in the patient’s biopsies (yellow outline) (Fig. 2, C and D). In patient lesions 2 and 3, we also documented areas of necrosis (red) and sclerosis (light blue) (Fig. 2 D). Lesion 2 was prominently composed of necrotic tissue, especially in the dermis, with evidence of parakeratosis, which signifies increased epidermal turnover at some point in the past (Fig. 2, C and D). Lesion 2 also had elevated scattered lymphohistiocytic inflammation (Fig. 2 D).

Lesion 3, which had some necrotic and sclerotic tissue, showed enhanced and prominent inflammatory infiltration, composed mostly of lymphocytes but also some plasma cells. Of note, there were no neutrophils detected in any of the control nor patient biopsies (Fig. 2 D).

Given the need to understand the pathophysiology in the patient’s lesions, we used spatial transcriptomics (ST) to uncover cellular pathways active in the patient’s skin lesions and the associated fibrosis as compared to healthy skin samples. Four 10-μm-thick Formalin-Fixed Paraffin-Embedded** (**FFPE) sections were analyzed: three derived from the patient (lesions 1–3) and one from a healthy donor (control 1). In addition, ST data from two independent healthy skin biopsies obtained from the GSE202011 repository were also included as controls (controls 2 and 3) (32).

**Figure 3.**
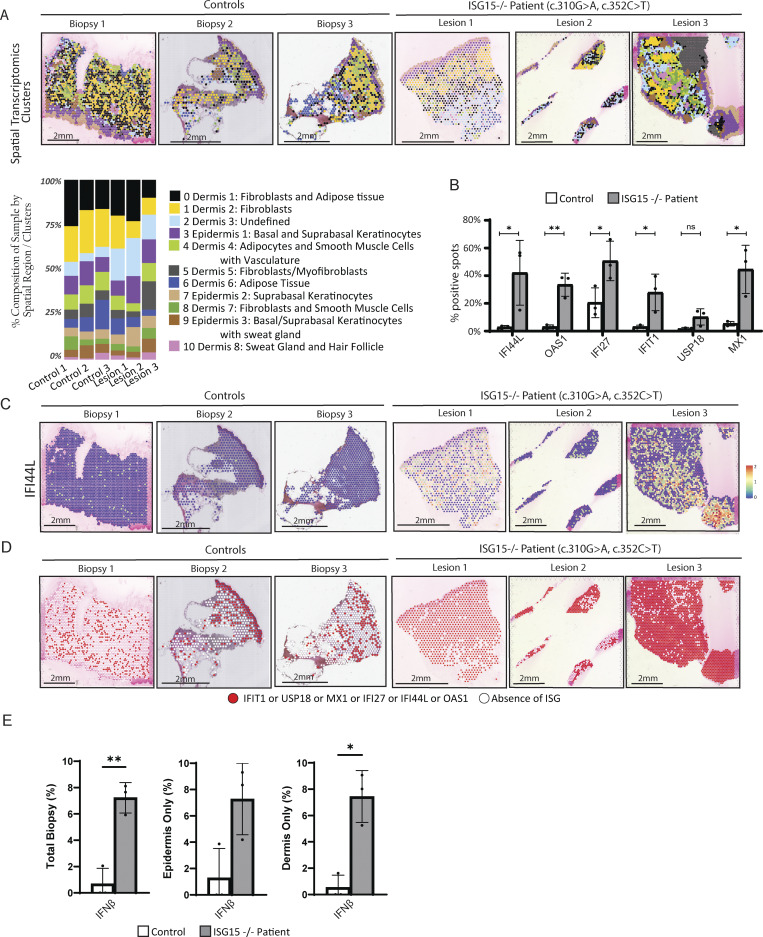
**IFN-I inflammation in patient skin biopsies. (A)** DEGs for each cluster were used to manually annotate the different clusters and then spatially visualized with one representative control (top). Percent composition of each cluster was calculated per biopsy (bottom). **(B)** Percentage of spots out of total biopsy that express each of the mentioned ISGs. **(C)** Localized expression and intensity levels of *IFI44L*. **(D)** Spatial expression of various ISGs. Red spots express at least one of the following ISGs: *IFIT1*, *USP18*, *MX1*, *IFI27*, *IFI44L*, or *OAS1*. White spots represent spots that do not express any of those ISGs. **(E)** Percentages of spots out of total biopsy, epidermis only, and dermis only that express *IFNB1*. P values were calculated with two-tailed *t* test. *P < 0.05; **P < 0.01.

**Figure S1.**
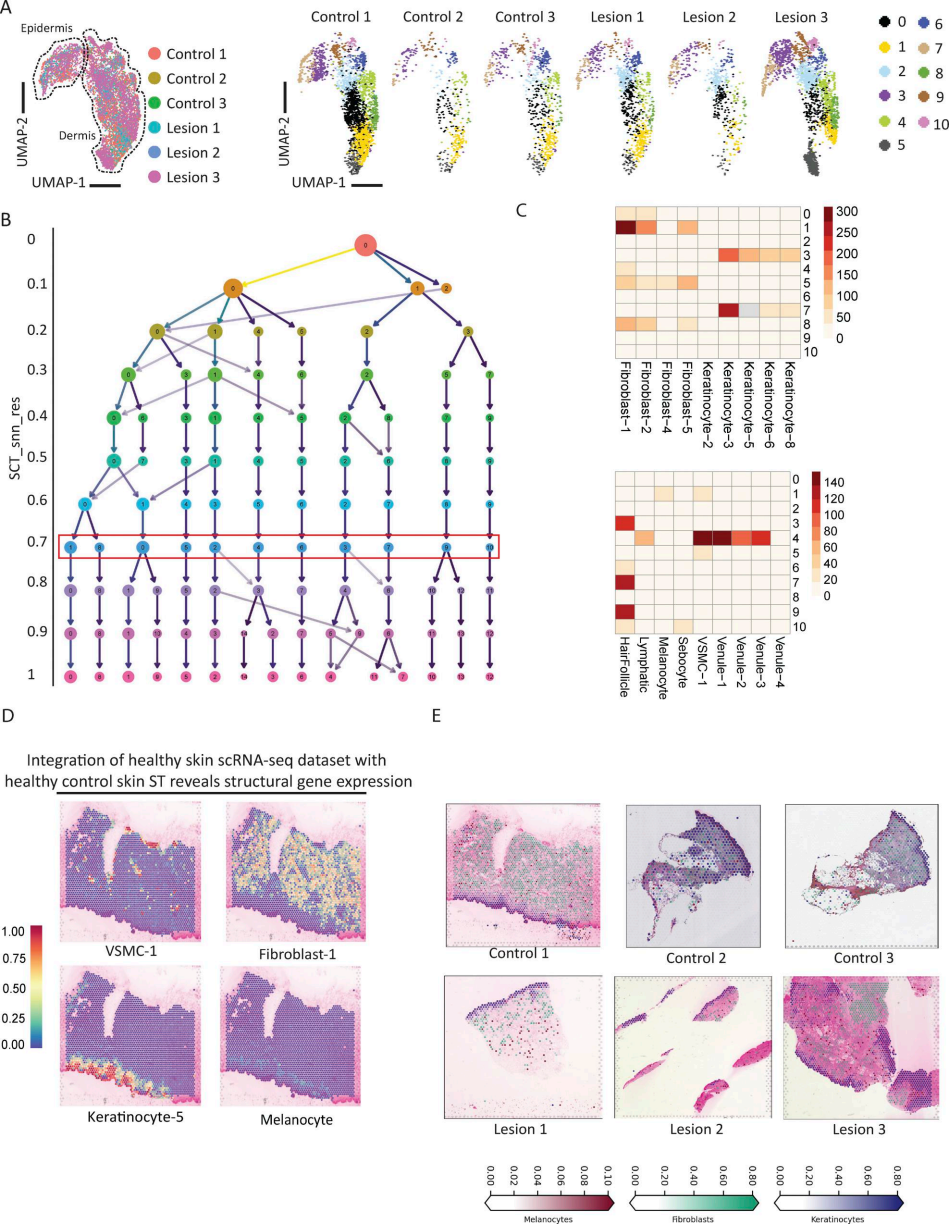
**ST** **deconvolution and cluster validation. (A)** Left: UMAP grouped by sample with defined epidermis and dermis areas. Right: UMAP grouped by cluster and biopsy. **(B)** Clustree to identify ideal cluster resolution. The red box indicates chosen resolution (0.8). **(C)** Multimodal integration analysis (MIA) showing the relative expression of structural cell types (top) and immune (bottom) clusters from an scRNA study in each of our ST cluster. **(D)** SpatialFeaturePlot showing the expression of the clusters annotated as VSMC-1, Fibroblasts-1, Keratinocyte-5, and Melanocyte from an scRNA study integrated into our ST samples. **(E)** UniCell deconvolution of a representative control and three patient lesional biopsies.

**Figure S2.**
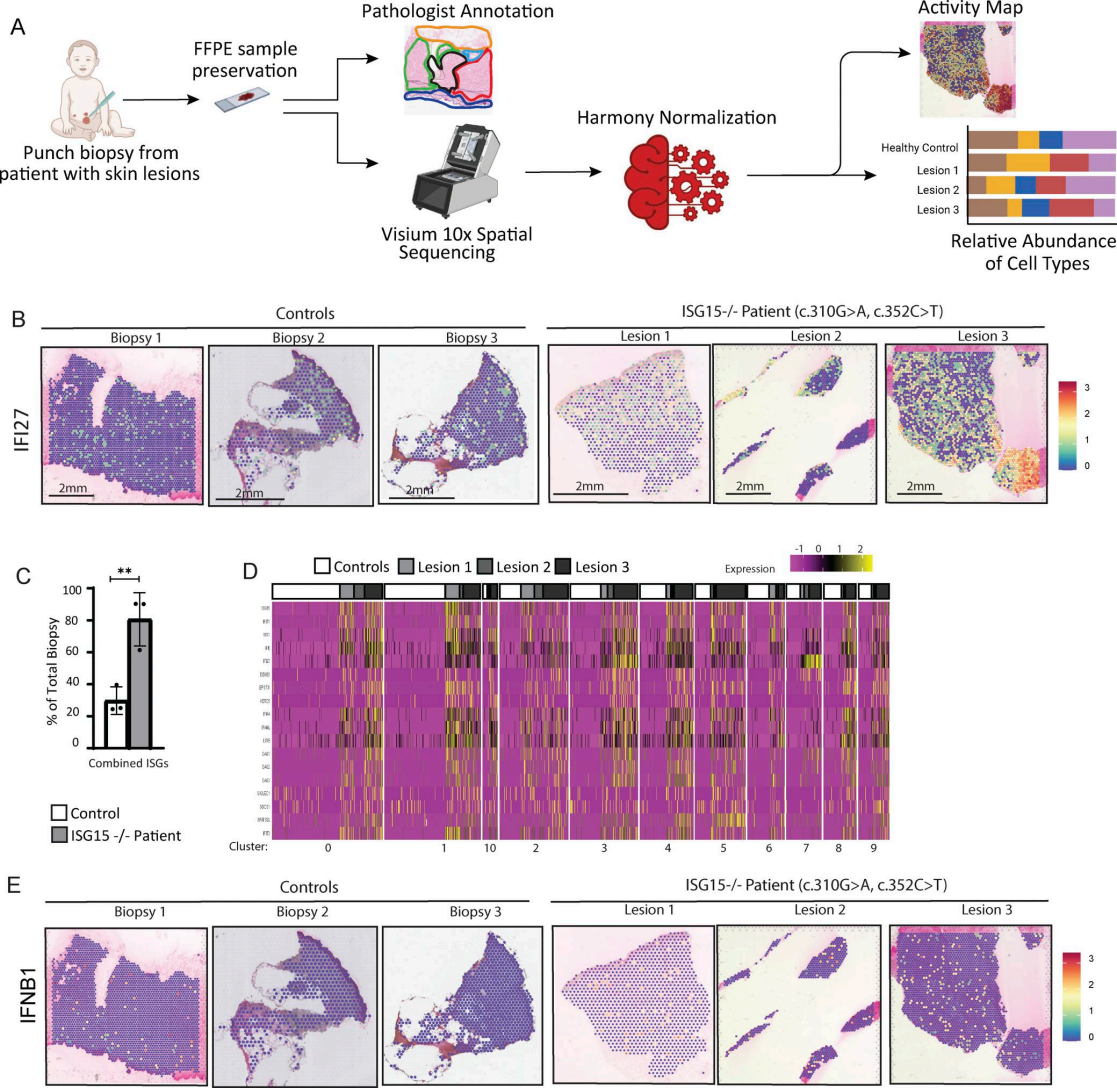
**Inflamed clusters revealed through ST. (A)** ST pipeline. **(B)** Spatial expression and intensity levels of *IFI27*. **(C)** Percentage of spots out of total biopsy expressing at least one of the six ISGs from Fig. 3 B. **(D)** Relative expression of various ISGs per cluster separated by sample biopsy. **(E)** Spatial expression and intensity levels of *IFNB1*. P values were calculated with two-tailed *t* test. **P < 0.01.

Following integration and Harmony normalization of the skin biopsies, we used principal component analysis (PCA), Uniform Manifold Approximation and Projection (UMAP), and manual annotation to generate distinct clusters and then calculated the percent contribution of each of those clusters for each biopsy (Fig. 3 A; and Figs. S1, A and B, S2 A). To validate our manual annotations, we performed multimodal integration analysis (MIA) using cell markers from an single cell RNA-sequencing (scRNA-seq) paper and found that they were highly concordant (Fig. S1 C). We additionally used UniCell deconvolution and integrated the annotated clusters from the aforementioned scRNA-seq paper into our samples to further validate the skin regions and cell types (Fig. S1, D and E).

As expected, we identified the typical skin layers, epidermis and dermis. Within the dermis, we noted the presence of fibroblasts and adipose tissue (cluster 0), fibroblasts (cluster 1), adipocytes with smooth muscle cells and vasculature (cluster 4), myofibroblasts (cluster 5), adipose tissue (cluster 6), fibroblasts with smooth muscle cells (cluster 8), and sweat glands with hair follicles (cluster 10). Within the epidermis, we noted the presence of basal and suprabasal keratinocytes (cluster 3), suprabasal keratinocytes (cluster 7), and basal/suprabasal keratinocytes with sweat glands (cluster 9). Notably, the cell-type composition was relatively comparable (Fig. 3 A). While the differentiation markers were stable across samples, we next wondered whether the skin lesions showed transcriptomics markers of inflammation.

Since ISG15-deficient patients are known to have elevated IFNα levels in their blood even in the absence of infection, we examined the presence of various ISGs (*IFIT1*,* USP18*,* MX1*,* IFI27*,* IFI44L*, and *OAS1*) (Fig. 3 B). In general, patient lesion spots were positive for at least one of six ISGs tested, with the integrated three controls showing <10% of positive spots (Fig. 3 B). Furthermore, *IFI44L* and *IFI27* were highly expressed in all three of the patient’s biopsies, in both the dermis and epidermis, as compared to the controls (Fig. 3 C and Fig. S2 B).

To identify the primarily inflamed regions in the skin, we created a SpatialFeaturePlot, highlighting spots that express at least one of the six ISGs in red and those that do not in white (Fig. 3 D). It was evident that the control biopsies did not exhibit substantial inflammation, with only about 25% of spots showing positivity for inflammation (Fig. S2 C). In contrast, the majority of spots in all three patient biopsies expressed at least one ISG: lesion 1 at 90%, lesion 2 at 61%, and lesion 3 at 90% (Fig. S2 C) with detectable levels in all skin layers (Fig. 3 D and Fig. S2 D). This first line of analyses allowed us to conclude that in ISG15 deficiency, all skin layers present with type I IFN–mediated inflammation signature.

Moreover, given that damaged cells, including keratinocytes, can secrete IFNβ, and since we were able to see in a previous study increased phosphorylation of STAT1 in this ISG15-deficient patient through immunohistochemistry (IHC) staining (a sign of excessive IFN-I signaling) (23), we sought to investigate whether IFN-I was also produced locally at the site of these chronic lesions. Indeed, we were able to detect a significant increase in *IFNB* transcripts in the patient’s total biopsy and dermis, with a still visible trend toward higher transcriptional levels in the epidermis (keratinocytes) (Fig. 3 E, Fig. S2 E).

### Activated macrophages could contribute to impaired wound healing

An inflammatory response triggered during an acute injury is a part of normal wound healing. However, prolonged inflammation can lead to pathological healing (33). Damage-associated molecular patterns, among other proinflammatory mediators, can activate the resident immune cells (i.e., macrophages), as well as recruit inflammatory cells, such as neutrophils and monocytes, from the circulation to the site of injury through the release of chemokines (33, 34). Such chemokines secreted by resident macrophages include CXCL1 and CXCL2 for neutrophil recruitment and CCL2 and CXCL12 for monocyte recruitment (35, 36). Neutrophils, which are the most abundant leukocytes in circulation, are the first responders following injury and contribute to the recruitment of circulating monocytes (35, 37), followed by T cell infiltration in the later inflammatory phase (38, 39). During an acute wound, macrophages exist in a proinflammatory state, referred to as M1 macrophages, which aid in the proper clearing of pathogens and/or damaged tissue. Once the clearance is completed, usually within days, the macrophages transition into an anti-inflammatory state (M2 macrophages) to allow the removal of immune cells and consequent wound healing (40). In the event of a chronic wound, proinflammatory M1 macrophages can remain active and do not transition to anti-inflammatory M2 macrophages, further contributing to the inflammation at the site of injury, and prevent wound healing, eventually leading to scar formation (40).

**Figure 4.**
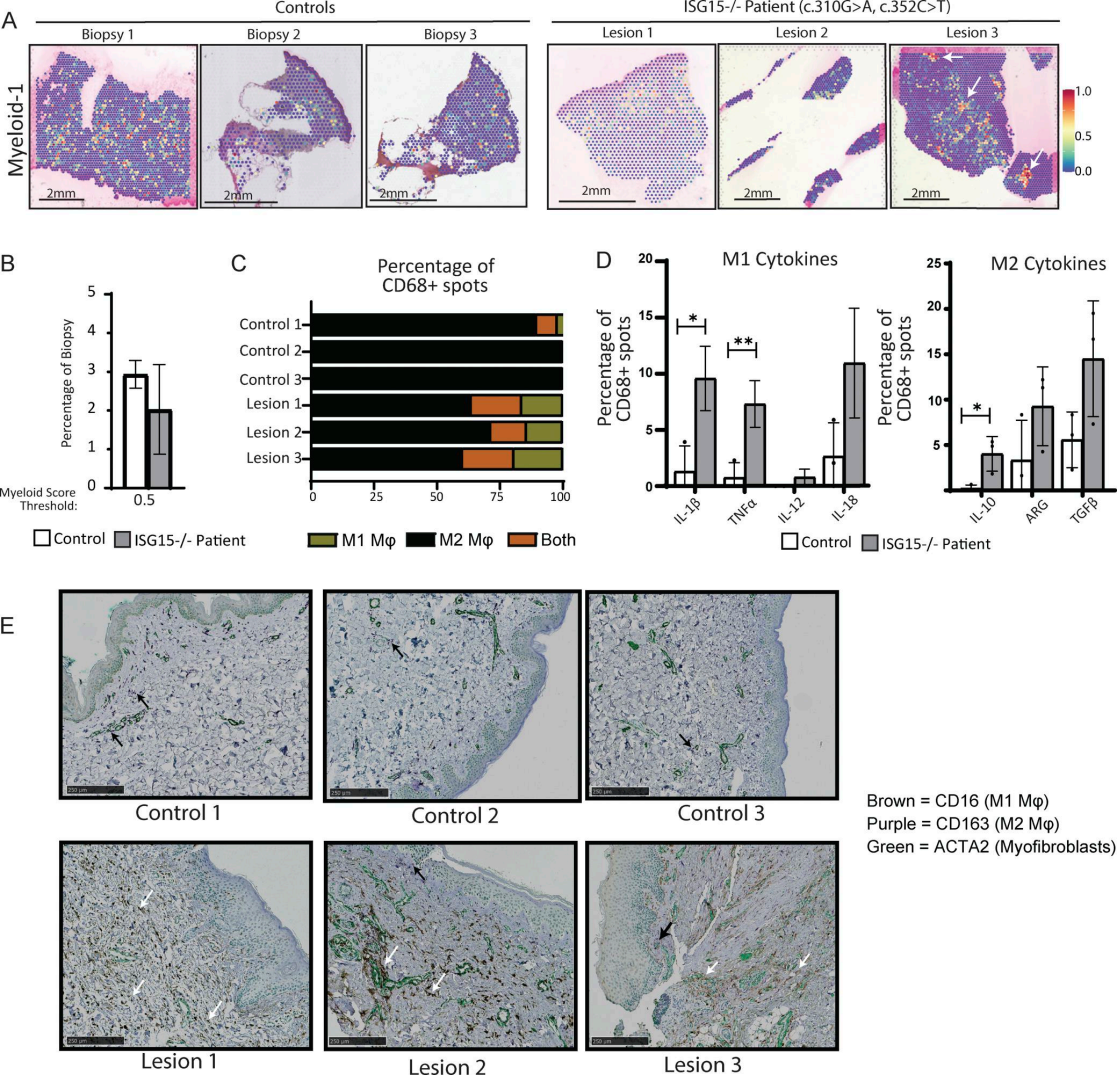
**Macrophage activation profile. (A)** SpatialFeaturePlot showing the expression of the myeloid cluster from an scRNA study integrated into our ST dataset. White arrows indicate myeloid hot spots. **(B)** Percentage of spots out of total biopsy above myeloid-probability score of 0.5 (50%) as defined from the scRNA Myeloid-1 Cluster. **(C)** Percent contribution of spots expressing M1 macrophage (Mφ) markers (green), M2 macrophage markers (black), or both M1 and M2 markers (orange) within the CD68^+^ spots. **(D)** Left: percentage of CD68^+^ that express M1 cytokines (*IL-1β*, *TNFα*, *IL-12*,* IL-18*). Right: percentage of CD68^+^ that express M2 cytokines (*IL-10*,* ARG*,* TGFβ*). **(E)** Multiplex IHC for CD16 (M1 macrophages), CD163 (M2 macrophages), and ACTA2 (myofibroblasts). White arrows indicate M1 macrophages (CD16); black arrows indicate M2 macrophages (CD163). P values were calculated with two-tailed *t* test. *P < 0.05; **P < 0.01.

Starting from that knowledge, we wanted to assess the numbers and quality of myeloid cells in the skin of the patient as compared to healthy skin samples. As earlier, we integrated a healthy skin scRNA-seq dataset with our ST data to identify the presence of myeloid cells indicated in the yellow-to-red scale (Fig. 4 A). There was no substantial difference in the percentage of positive spots between control and patient’s biopsies when the threshold was set to 0.5; however, the distribution of the positive spots in lesion 3, which was sampled during an active wound, had distinct myeloid hot spots (denoted by white arrows) (Fig. 4, A and B).

**Figure S3.**
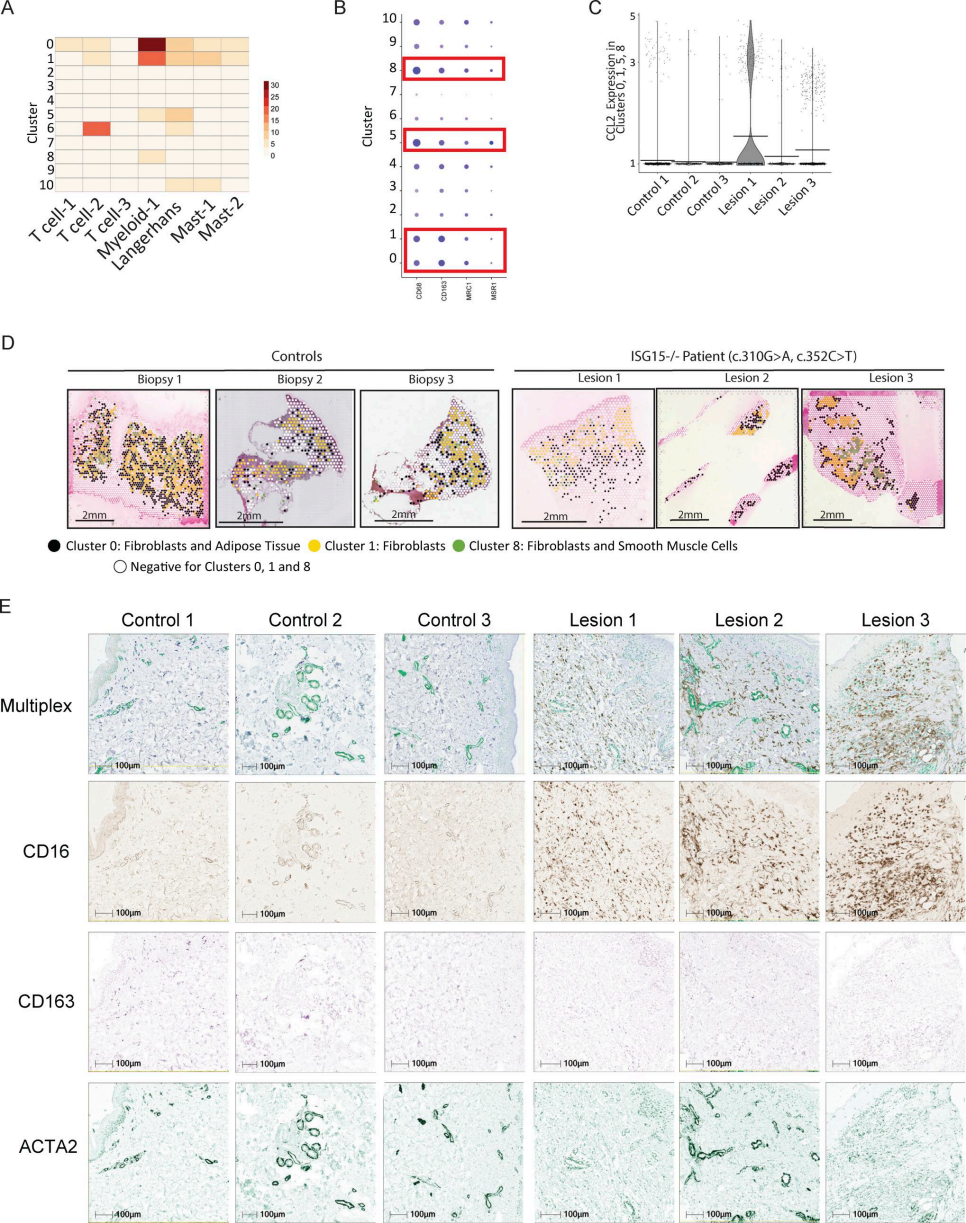
**Myeloid compartment validation. (A)** Multimodal integration analysis (MIA) showing the relative expression of immune cluster from an scRNA study in each of our ST cluster. **(B)** Average expression and percentage of spots expressing various macrophage markers in each cluster. **(C)** Expression levels of *CCL2* in clusters identified to be positive for monocytes (clusters 0, 1, 5, and 8). **(D)** Spatial location of myeloid-positive clusters (0, 1, and 8) depicted in black, yellow, and green, respectively, and the absence of those clusters depicted in white spots. **(E)** Deconvoluted multiplex IHC for CD16, CD163, and ACTA2.

Infiltration by the myeloid compartment was further validated by the identification of macrophage markers CD68, CD163, MRC1, and MSR1 in the clusters (Fig. S3, A and B), as well as the presence of increasing production of CCL2, a monocyte chemoattractant, in those dermal clusters (Fig. S3C). This could be hinting to the possibility of myeloid cell infiltration in the dermis layer of the skin to clear the active wound (Fig. 4 A; and Fig. S3, A and D).

We then sought to assess differences in M1-M2 macrophage polarization across our biopsies. We looked within the CD68^+^ spots that expressed at least one of the M1 markers (CD163 or ARG1) or M2 markers (FCGR3A or FCGR1A). While macrophage polarization consisted almost entirely of M2 macrophages in the control biopsies, there was a significant contribution of M1 macrophages in all three lesional biopsies (Fig. 4 C), as well as much higher percentage of spots expressing both M1 and M2 markers (Fig. 4 C).

Lastly, to assess the activity of those macrophages, we quantified the number of spots within the CD68^+^ spots that expressed M1 and M2 cytokines. As expected, we saw elevated transcriptional levels of proinflammatory M1 cytokines (*IL1β*, *TNFα*, *IL12*, and *IL18*) in the patient’s lesions (Fig. 4 D). Interestingly, we also saw elevated transcriptional levels of M2 cytokines (*IL10*, *ARG*, and *TGFβ*) in patient’s biopsies as compared to the healthy control (Fig. 4 D). This could indicate that even though M2 macrophages are present in the control and during a healthy state, they are not necessarily as activated. In a chronic wound state, there might be a functional activity of both M1 and M2 macrophages in maintaining an active wound, propagating damage, and stalling proper wound healing.

To further validate the differences in macrophage polarization that we observed through ST between the healthy controls and patient’s biopsies, we performed multiplex IHC staining for CD16 (a marker primarily associated with M1 proinflammatory macrophages) and CD163 (a highly specific M2 macrophage marker) (Fig. 4 E) (41, 42, 43, 44, 45, 46, 47, 48). Although CD16 is a known marker for neutrophils, we did not detect the presence of neutrophils using either ST or H&E staining (49). CD16 is also expressed by NK cells; however, NK cell presence detected by ST was minimal (49). This allowed us to confidently exclude neutrophils and NK cells as the source of the CD16^+^ signal observed in our IHC. In contrast, ST revealed a prominent population of other myeloid cells, which correlated more closely with the distribution and abundance of CD16^+^ cells in our IHC. Taken together, these observations strongly suggest that the CD16^+^ cells are M1 macrophages.

Whereas the control biopsies showed some CD163 signal as indicated by the black arrows, they did not have any detectable CD16 (Fig. 4 E and Fig. S3 E). On the other hand, all three patient’s biopsies had very high levels of CD16^+^ cell infiltration (white arrows) with some CD163+ cells, albeit at lower levels (Fig 4 E and Fig. S3 E).

Seeing the elevated expression of TGFβ, we next wanted to explore inflammation and fibroblast activation of resident tissue cells.

### ISG15-deficient epithelial cells are poised for epithelial-to-mesenchymal transition (EMT)

Fibrosis is a reparative process involving the deposition of collagen to support wound healing. However, when fibrosis becomes excessive, it can replace normal tissue with connective tissue, leading to scar formation. While fibrosis is a complex cellular process, the primary drivers are myofibroblasts and fibrocytes. Myofibroblasts typically originate from resident fibroblasts at the injury site, which become activated and then deposit type III and type V collagen. These cells also increase the production of extracellular matrix remodelers, such as metalloproteinases. Additionally, myofibroblasts can arise through EMT and endothelial-to-mesenchymal transition (50).

Fibrocytes, on the other hand, are myeloid-derived cells that are recruited to the wound site within days, arriving more quickly than the slow-moving myofibroblasts. Although fibrocytes can produce collagen, their contribution in this regard is relatively minor. Instead, they play a more indirect role in fibrosis by secreting significant amounts of TGF-β. The lack of specific markers for fibrocytes renders their study in vivo challenging; however, the dual expression of CD45 and CD34 is generally used as an indicator of fibrocytes (51, 52, 53, 54, 55, 56). We were unable to identify any spots that expressed both CD45 and CD34 in any of the biopsies, and thus, we could not further assess fibrocyte activity if present.

**Figure 5.**
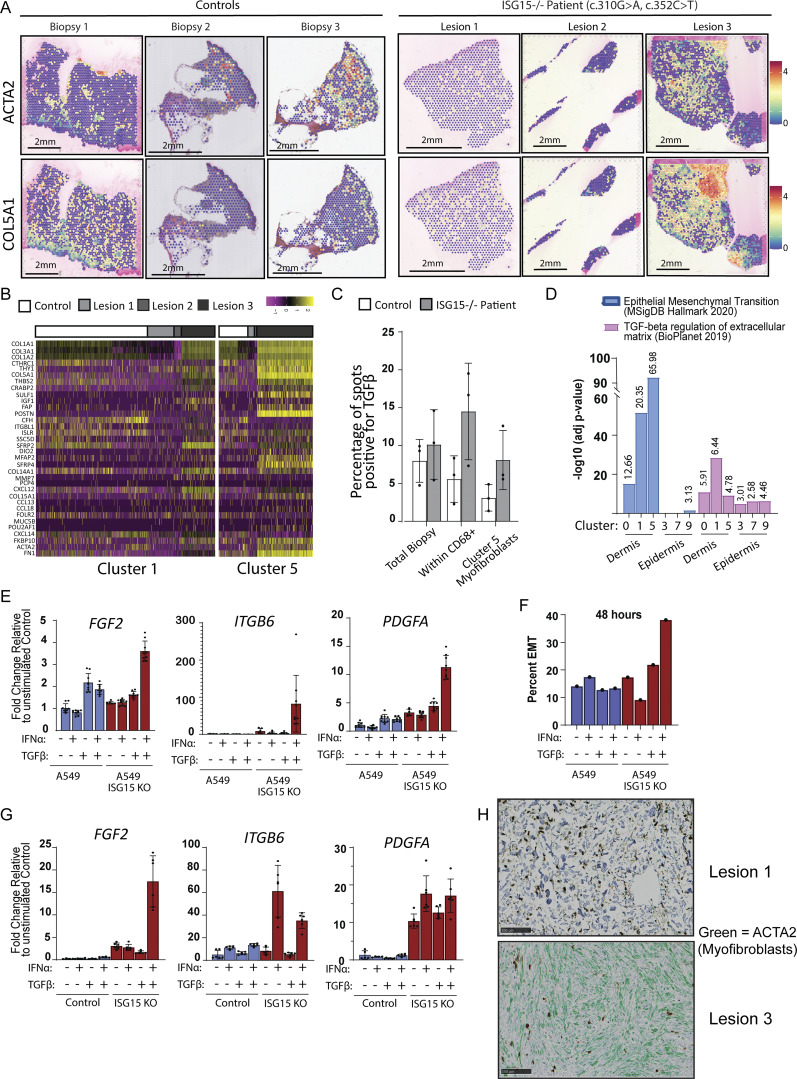
**Myofibroblast activation in ISG15-deficient cells. (A)** Spatial expression and intensity levels of two myofibroblast markers (*ACTA2* and *COL5A1*). **(B)** Relative expression of various myofibroblast markers for clusters 1 and 5 separated by sample biopsy. **(C)** Percentage of total spots that express *TGFβ* in the entire biopsy, within CD68^+^ spots only, and within myofibroblast cluster 5. **(D)** Significance of pathway enrichment for clusters 0, 1, 5, 3, 7, and 9 generated by MSigDB Hallmark 2020 and BioPlanet 2019 analysis from Enrichr. Adjusted P values are shown. **(E)** Relative RNA levels of myofibroblast markers (*PDGFA*, *ITGB6*, *FGF2*) following a 72-h treatment with 1,000 IU/ml IFNα2b, 10 ng/ml TGFβ, or both of WT and ISG15 KO lung epithelial cells (A549). **(F)** Percentage of cells undergoing EMT upon TGFβ/IFNα stimulation. **(G)** Relative RNA levels of myofibroblast markers (*PDGFA*, *ITGB6*, *FGF2*) following a 5-day treatment with 1,000 IU/ml IFNα2b, 10 ng/ml TGFβ, or both of WT and ISG15 KO fibroblasts. **(H)** ACTA2 (myofibroblasts) IHC for lesion 1 and lesion 3. P values were calculated with two-tailed *t* test. *P < 0.05.

**Figure S4.**
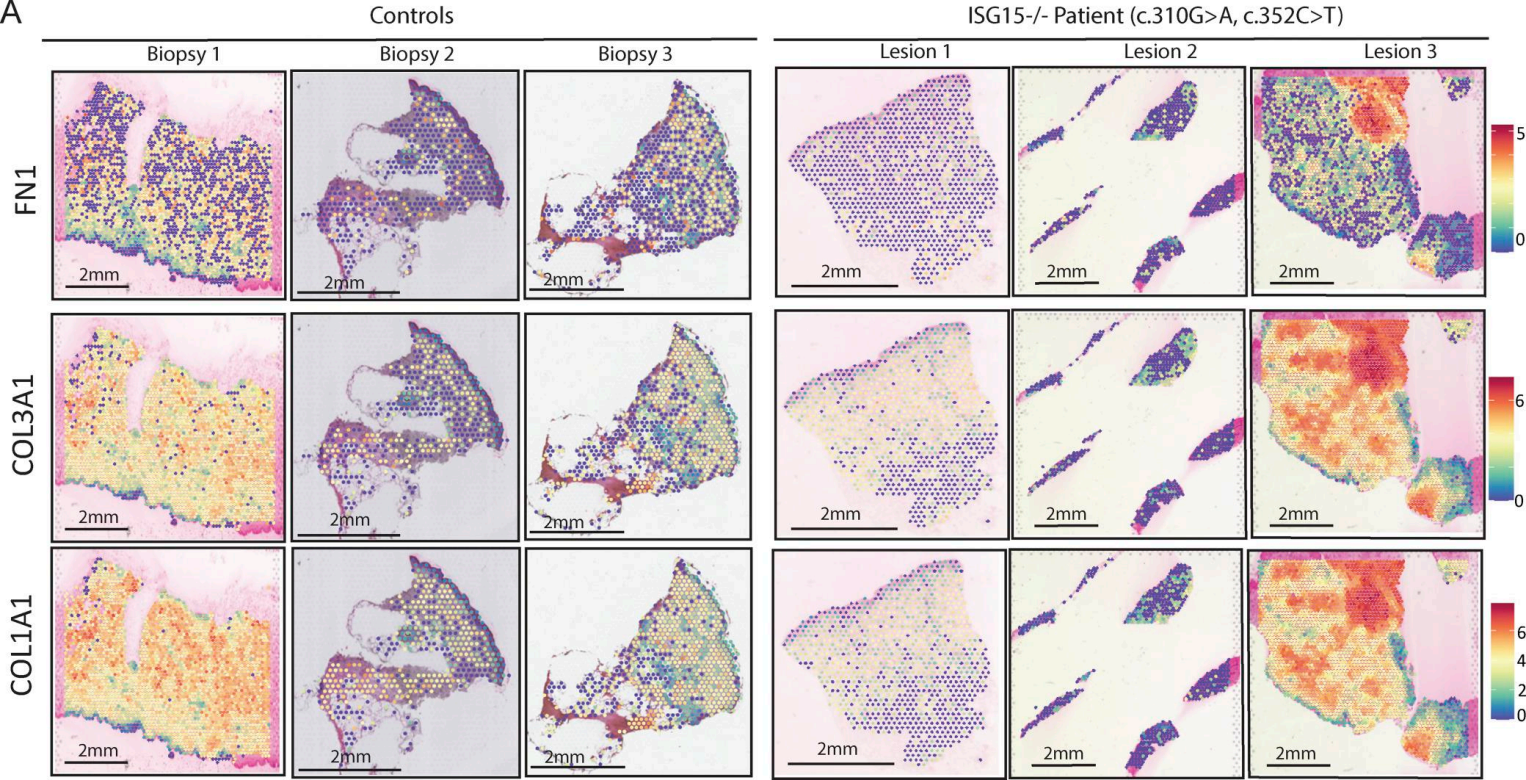
**Myofibroblast markers through ST. (A)** Spatial expression and intensity levels of various myofibroblast markers (*FN1*, *COL3A1*, and *COL1A1*).

We then assessed myofibroblast-associated transcriptional signature. *COL5A1* and *ACTA2* were significantly upregulated in lesion 3, which had an active wound at the time of collection (Fig. 5 A). This pattern was also observed for other myofibroblast markers, such as *FN1*, *COL1A1*, and *COL3A1* in lesion 3 (Fig. S4 A). In addition, we looked at the differentially expressed genes (DEGs) of our clusters, noting that fibroblasts from cluster 5 showed overexpression of a substantial number of myofibroblast markers. Fibroblasts in cluster 1 also expressed some myofibroblast markers but not to the same extent as cluster 5 (Fig. 5 B). Even though cluster 5 is present in the control biopsies, the myofibroblast markers were primarily elevated in cluster 5 of lesion 3 and not of healthy control (Fig. 3 A and Fig. 5 B).

TGFβ is the main driver for myofibroblast activation, and we have shown in Fig. 4 D that CD68^+^ spots have a high number of spots that express TGFβ, implying that they could be the initiators of fibrosis in ISG15-deficient patients. Moreover, once fibroblasts become myofibroblasts they secrete TGFβ that acts in an autocrine and paracrine fashion and in a positive feedback loop to maintain the myofibroblast phenotype, as well as to induce further transitioning of neighboring fibroblasts into myofibroblasts. Indeed, we noticed higher levels of TGFβ in clusters 5 (fibroblasts/myofibroblasts) further validating the presence and activity of myofibroblasts (Fig. 5 C).

**Figure S5.**
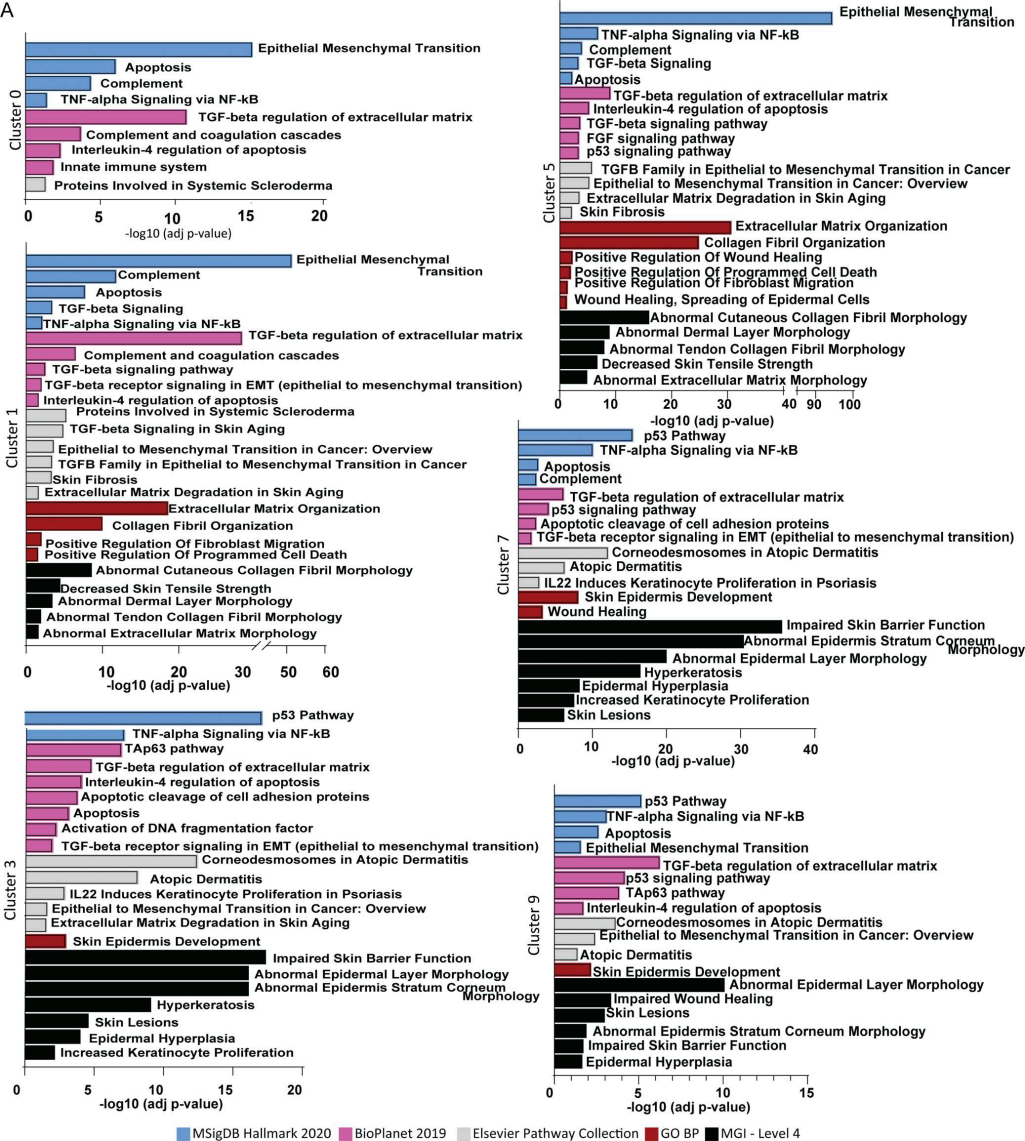
**Pathway enrichment analysis. (A)** Significance of pathway enrichments for clusters 0, 1, 3, 5, 7, and 9 generated by MSigDB Hallmark 2020 (blue), Gene Ontology Biological Processes (GO BP; red), MGI Level 4 (black), BioPlanet 2019 (pink), and Elsevier Pathway Collection (gray) analysis from Enrichr. Adjusted P values are shown. TGF-beta receptor signaling in EMT (epithelial to mesenchymal transition). IL22 Induces Keratinocyte Proliferation in Psoriasis.

We proceeded with performing pathway enrichment on the DEG of cluster 5 (myofibroblasts) using different tests. MSigDB Hallmark 2020 showed significant contribution of markers, such as *ITGB1*, *COL16A1*, *POSTN*, and *ACTA2*, involved in EMT; Gene Ontology Biological Processes showed enhanced extracellular matrix organization markers; and BioPlanet 2019 and Elsevier Pathway Collection showed induction of TGFβ signaling, further supporting the presence and activity of myofibroblasts in cluster 5. Interestingly, MGI Level 4 (Mouse Genome Informatics Mammalian Phenotype Level 4) showed elevated markers associated with a number of different skin conditions, such as abnormal cutaneous collagen fibril morphology, decreased skin tensile strength, and abnormal dermal layer morphology, all of which could contribute to fibrosis (Fig. 5 D and Fig. S5 A).

Given the proximity of epidermal clusters 3, 7, and 9 to the two dermal clusters that are predominantly constituted by macrophages (clusters 0 and 1), as well as to their close proximity to the myofibroblasts (cluster 5), we evaluated TGFβ signature in those regions as well. Pathway enrichment analysis revealed a TGFβ regulation of extracellular matrix signature, with epidermal cluster 9 showing an EMT profile as well (Fig. 5 D and Fig. S5 A).

To recapitulate this in vitro, we treated WT and ISG15 knockout (KO) A549 lung epithelial cells with either TGFβ or IFNα, or both cytokines combined for 3 days and then assessed myofibroblast marker levels (*PDGFA*, *ITGB6*, and *FGF2*) through qPCR. TGFβ alone or with IFNα induced a small increase of *PDGFA* and *FGF2* mRNA levels in the control cell line (Fig. 5 E). Interestingly, combined IFNα and TGFβ treatment of ISG15 KO A549 cells induced significantly higher levels of *PDGFA*, *ITGB6*, and *FGF2* mRNA compared with WT cells (Fig. 5 E). In addition to those traditional markers, we also assessed EMT of A549 by looking at the morphological changes of cells upon cytokine treatments through imaging (57). Following cell segmentation with CellPose, we used 5,000 μm^2^ as the threshold for EMT, and we noticed that individual treatments (either IFNα or TGFβ) did not have major effects on either WT or ISG15 KO cells; however, dual treatment did induce a significant increase in ISG15 KO cell size with ∼40% of the cells having an area of >5,000 μm^2^ 48 h after treatment (Fig. 5 F).

A similar response to A549 cells was also observed in fibroblasts lacking ISG15. Treatment with IFNα and TGFβ caused a significant induction of *FGF2* in ISG15 KO fibroblasts compared with the control. Moreover, either IFNα or IFNα/TGFβ treatment also induced *ITGB6* and *PDGFA* at a higher degree than in WT (Fig. 5 G).

Lastly, we also verified the presence of myofibroblasts in our patient’s biopsies by assessing the levels of smooth muscle actin (*ACTA2*) through IHC. All biopsies had *ACTA2* in follicular structures, vessels, and the basal layer of the epidermis (Fig. 4 E). However, in agreement with our ST, lesion 1 and lesion 3 showed enhanced *ACTA2* and thus activated myofibroblasts in the biopsies (Fig. 5 H and Fig. S3 E).

### Spatial transcriptomics reveals elevated levels of apoptotic markers in ISG15-deficient skin dermis

Given the presence of ulcerating and necrotic plaques in patients with ISG15 deficiency, as well as the observed IFN-I–induced apoptosis in vitro (see below), we sought to investigate the presence of elevated apoptotic or necroptotic markers in our ST dataset. Apoptosis is a biological process that removes dysfunctional, damaged, or infected cells in a regulated fashion in response to certain signals. Therefore, apoptosis is crucial not only in tissue homeostasis, but also in tissue health, repair, and maintenance (58, 59, 60, 61). Apoptosis leads to cell shrinkage, cell fragmentation, and subsequent removal of the dying cell by phagocytic cells, such as macrophages, and there are two main pathways—the extrinsic and the intrinsic—that can activate this process (62, 63, 64). Extracellular ligands, such as TNFα and FasL, can trigger the extrinsic arm of apoptosis by binding to their receptors on the surface of the cells. This activates initiator caspases, such as caspase 8, which subsequently activate effector caspases such as caspase 3 (65, 66, 67, 68). On the other hand, intracellular stressors, such as DNA damage and replication fork stalling, activate the intrinsic arm of apoptosis, which is regulated by the proapoptotic proteins Bax and Bak, with the subsequent release of cytochrome c from the mitochondria (69, 70, 71).

Necroptosis is a different type of programmed cell death that differs morphologically and biochemically from apoptosis (72). Necroptosis exerts its function through the activation of RIPK1 and RIPK3. TNFα can also induce necroptosis in the absence of checkpoints like TBK1 (73) through the activation of RIPK1/RIPK3, which leads to the activation of MLKL. MLKL then translocates to the cell membrane causing the rupture of the membrane through the formation of pores (71, 74, 75).

**Figure 6.**
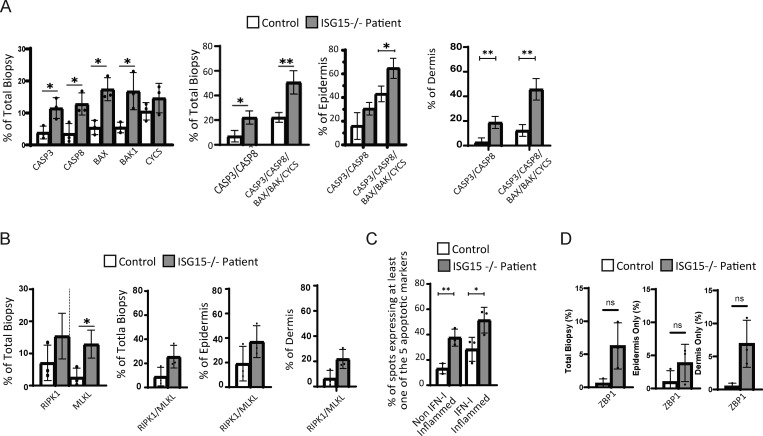
**Cell death markers identified through ST. (A)** Left: percentage of spots expressing apoptosis markers (*CASP3*, *CASP8*, *BAX*, *BAK1*, and *CYCS*) out of total biopsy. Middle and right: percentage of spots expressing either two or five apoptosis markers in total biopsy, epidermis only, or dermis only (respectively). **(B)** Left: percentage of spots expressing necroptosis markers (*RIPK1* and *MLKL*) out of total biopsy. Middle and right: percentage of spots expressing either RIPK1 or MLKL in total biopsy, epidermis only, or dermis only (respectively). **(C)** Percentage of spots expressing at least one apoptotic marker (*CASP3*, *CASP8*, *BAX*, *BAK1*, or *CYCS*) that also express or not at least one of the ISGs (*IFIT1*, *USP18*, *MX1*, *IFI27*, *IFI44L*, or *OAS1*). **(D)** Percentage of spots out of total biopsy, epidermis only, and dermis only that express *ZBP1*. P values were calculated with a two-tailed *t* test. *P < 0.05; **P < 0.01.

We were able to detect a pronounced transcriptional induction of apoptotic markers (*CASP3*, *CASP8*, *BAX*, *BAK1*, *CYCS*) in the patient’s biopsies alongside less prominent markers of necroptosis (*RIPK1* and *MLKL*) as compared to nonlesional skin (Fig. 6, A and B). Upon examining these markers individually, we observed that all apoptotic markers were elevated in all three lesions compared with the average of the control biopsies (Fig. 6 A). If we looked at two or five markers combined, the difference between control and lesional skin was even more pronounced (Fig. 6 A). To account for the varying ratios of epidermis to dermis in the six biopsies, we analyzed the percent expression of the two marker groups within each tissue layer. Interestingly, when epidermis was examined alone, there was no significant difference in apoptotic marker expression between the lesions and the control, suggesting the dermis layer drives the majority of the cell death signature (Fig. 6 A). The consistently elevated death markers in the epidermis of both control and patient biopsies could derive from the high turnover of keratinocytes in the skin. These results combined suggested relatively stable skin layer and skin cell-type composition, with most tissue damage affecting the dermis and stemming from apoptosis.

We then investigated dermis-layer inflammation in greater depth. Spots that expressed at least one of the six ISGs mentioned above (*IFIT1*, *USP18*, *MX1*, *IFI27*, *IFI44L*, or *OAS1*) were referred to as IFN-I inflamed. When we considered only the IFN-I inflamed spots and looked for positivity for *CASP3*, *CASP8*, *BAX*, *BAK1*, or *CYCS*, the percentage of spots expressing at least one of those apoptotic markers was further augmented in IFN-I inflamed compared with noninflamed spots (Fig. 6 C). Irrespective of apoptosis, prominently high ISG expression was documented across all clusters of the lesional biopsies but not of the control biopsies (Fig. 3 D and Fig. 6 C). In addition, we were able to see a trend, although not significant, toward the elevated expression of *ZBP1* (an IFN-inducible cytosolic nucleic acid sensor and a major driver of PANoptosis through the RIPK1–RIPK3–caspase 8 axis) in the dermis of the patient’s biopsies (Fig. 6 D). Overall, these data suggest that skin is affected by circulating IFN-I, and prominently so across the lesions where apoptosis is also pronounced.

### ISG15-deficient fibroblasts display enhanced IFN-I–induced cell death and antiproliferative properties

**Figure 7.**
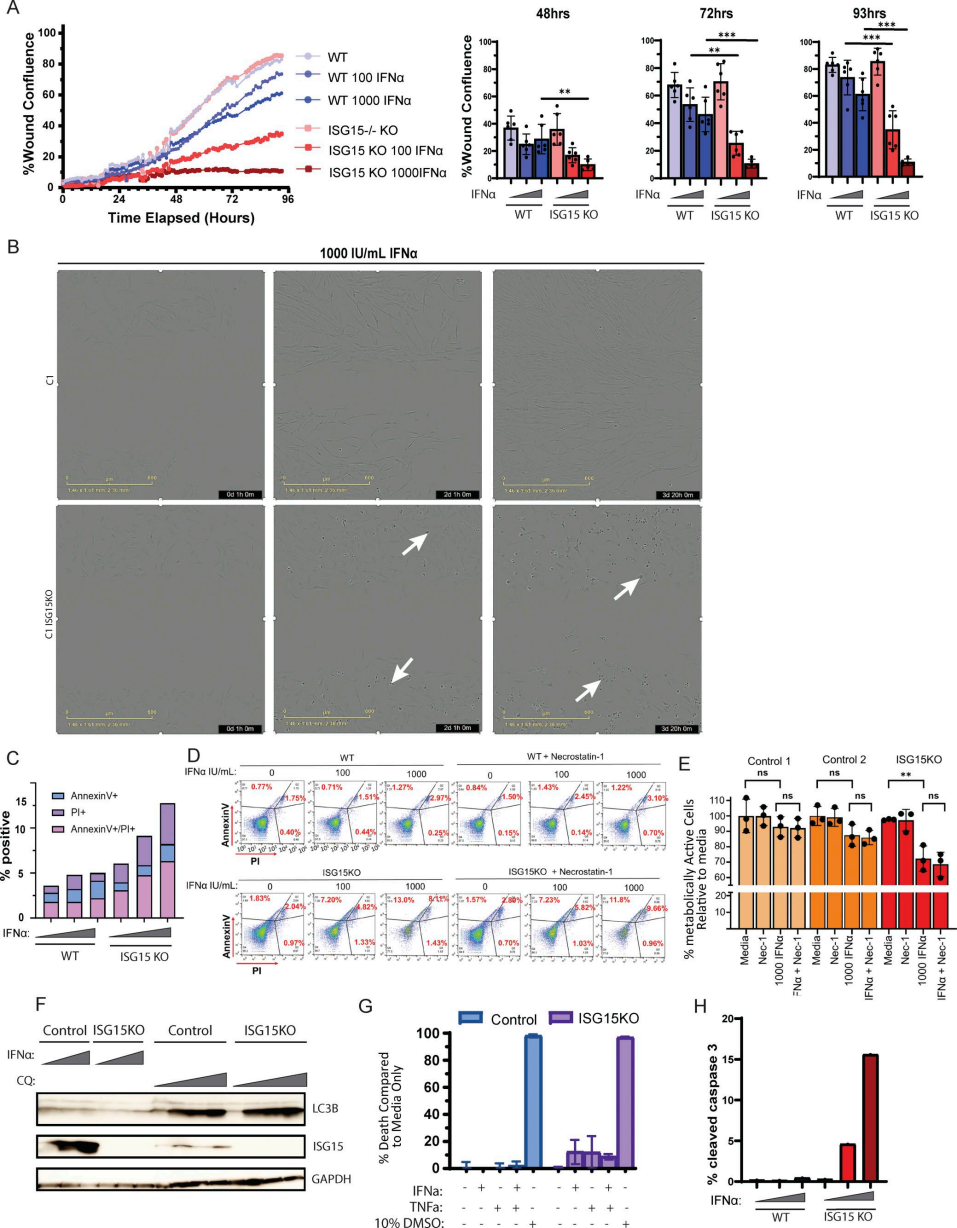
**IFNα induces apoptosis in ISG15 KO cells. (A)** Healing/migration ability of WT and ISG15 KO fibroblasts was assessed with the scratch assay. Left; percent confluency was recorded every hour for a total of 93 h. Right; experimental replicates at 48, 72, and 93 h after scratch. **(B)** Representative images of WT and ISG15 KO fibroblasts at 1 h (left), 49 h (middle), and 92 h (right) of 1,000 IU ml^−1^ IFNα treatment. White arrows indicate macroscopic evidence of cellular stress. **(C)** WT and ISG15 KO fibroblasts were treated with IFNα2b (0, 100, 1,000 IU/ml) for 72 h, and cell death was quantified by measuring Annexin V and Propidium Iodide (PI) levels through flow cytometry. **(D)** Control and ISG15 KO fibroblasts were treated with IFNα2b (0, 100, 1,000 IU/ml) for 72 h following a 30-min pretreatment with necrostatin-1, and cell death was quantified by measuring Annexin V and PI levels through flow cytometry. **(E)** Metabolic activity of two control and one ISG15 KO fibroblast cell lines was assessed with the Deep Blue assay. Nec-1; necrostatin-1. **(F)** Control and ISG15-deficient fibroblasts were treated with either IFNα2b (1, 100, 1,000 IU/ml) or chloroquine (0, 25, 50, 100 μM) for 72 h. Cell lysates were analyzed by western blotting for autophagy (LC3B). **(G)** Control and ISG15-deficient fibroblasts were treated with either IFNα2b (100 IU/ml), or TNFα (5 ng/μl), or both for 72 h, and percent death was assessed with the Deep Blue assay as compared to the nontreated condition for each variant. **(H)** WT and ISG15 KO fibroblasts were treated with IFNα2b (0, 100, 1,000 IU/ml) for 72 h, and cell death was quantified by measuring cleaved (active) caspase 3 levels through flow cytometry. Source data are available for this figure: SourceData F7. P values were calculated with two-tailed *t* test. **P < 0.01; ***P < 0.001.

IFN-Is are known to have antiproliferative and proapoptotic properties (76, 77, 78, 79), and they have been shown to induce apoptosis in ISG15 KO induced pluripotent stem cells (iPSC)-derived macrophages (80). Therefore, we sought to test how IFN-Is may affect the tissue healing in the context of ISG15 deficiency, where individuals have a continuous unchecked IFN-I signaling (23, 81). We first assessed the ability of ISG15 KO fibroblasts to migrate and repopulate an artificial wound in vitro by performing a scratch assay. An equal number of fibroblasts (WT and ISG15 KO) were seeded the day before, then pretreated with 0, 100, or 1,000 IU/ml of IFNα2b for 6 h. The scratch was introduced by a pin, and the cells were treated again with the same amount of IFNα2b. Images were taken every hour for 4 days (Fig. 7, A and B). In the absence of IFNα, control and ISG15 KO fibroblasts proliferated at similar rates, reaching a wound confluency of 83% and 85%, respectively, by the end of day 4. Observable differences in wound repopulation rates between the control and ISG15 KO fibroblasts were evident as early as 48 h after scratch introduction and stark by 96 h, where WT cells reached a confluency of about 60%, while ISG15-deficient fibroblasts reached only 10% confluency (Fig. 7, A and B). IFNα greatly affected the cell’s ability to proliferate and repopulate the artificial wound in a dose-dependent manner, with a much greater effect on ISG15 KO fibroblasts compared with WT. Moreover, ISG15 KO fibroblasts showed visible cellular stress upon prolonged IFNα treatment, as indicated by the evident morphological changes such as membrane blebbing (Fig. 7 B).

To assess whether this decrease in the migration capacity of ISG15 KO cells upon IFN treatment was purely due to the antiproliferative properties of IFNα, or whether cell death was involved, we assessed various cell death pathways in our in vitro system. A 72-h treatment of fibroblasts with 100 or 1,000 IU/ml of IFNα2b revealed a dose-dependent cell death of ISG15 KO fibroblasts. No significant increase in cell death was observed for the WT fibroblasts (Fig. 7 C). To decipher the cell death pathway that could be contributing to this increased cell death of the KO line, we looked to PANoptosis, necroptosis, autophagy, and apoptosis. Neither necroptosis nor autophagy was observed in our in vitro experiments (Fig. 7, D–F). As shown previously in Fig. 6 D, the expression of *ZBP1* was slightly elevated in the biopsies; however, treatment with necrostatin-1, a necroptosis/RIPK1 inhibitor, together with IFNα2b did not reverse the level of cell death (Fig. 7 D), nor the loss of metabolic activity of the ISG15 KO cells (Fig. 7 E). There were no significant differences in the levels of LC3B, a marker of autophagy, upon treatment with IFNα2b for neither the WT nor the ISG15 KO fibroblasts (Fig. 7 F). Response to other triggers, such as TNFα, was also investigated. There was no noticeable effect of TNFα on cell death or metabolic activity, and when a dual treatment of IFNα2b with TNFα was administered, it did not exacerbate the IFN-I effect (Fig. 7 G).

We then assessed the levels of active caspase 3, an apoptosis marker. We observed a dose-dependent response in active caspase 3 in the ISG15 KO fibroblasts, reaching levels of ∼15%, whereas the control fibroblasts were not affected (Fig. 7 H).

**Figure 8.**
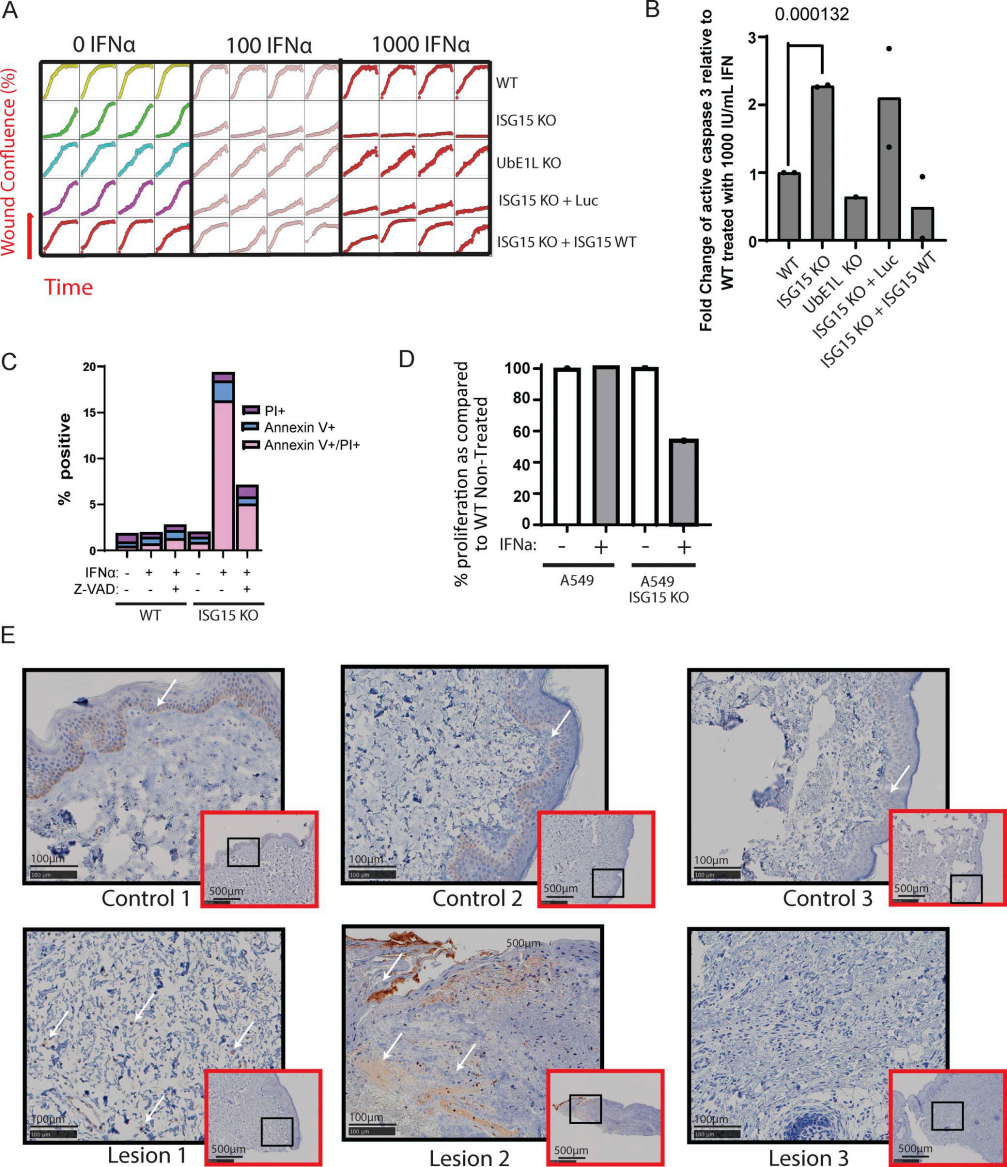
**Reconstitution with WT ISG15 and inhibition of apoptosis rescue apoptosis of IFNα-treated ISG15 KO cells. (A)** Healing/migration ability of WT, UBE1L KO, and ISG15 KO fibroblasts, as well as ISG15 KO cells complemented with WT ISG15 or Luc, was assessed with the scratch assay for 93 h. **(B)** Apoptosis following 72-h IFNα2b treatment of WT, UBE1L KO, and ISG15 KO fibroblasts, as well as ISG15 KO cells complemented with WT ISG15 or Luc, was assessed by quantifying cleaved caspase 3 levels through flow cytometry. **(C)** Rescue of A549 ISG15 KO IFNα2b-induced cell death with pan-caspase inhibitor (Z-VAD). **(D)** Proliferation of ISG15 KO lung epithelial cells (A549) with or without IFNα2b as compared to control. **(E)** CASP3 IHC for three healthy volunteer and three patient’s skin biopsies. White arrows indicate areas of cleaved Caspase-3 (brown).

When we reintroduced the ISG15 WT gene back into the ISG15 KO fibroblast cell line, the phenotype was rescued. The ability of the cells to migrate and repopulate the artificial wound of a scratch assay was fully restored as indicated by the similar levels of wound confluence between WT and ISG15 KO + WT ISG15 lines. Moreover, the introduction of Luc (ISG15 KO + Luc) did not reverse the ISG15-deficient phenotype, and no defect was observed in ISGylation-deficient cells (UBE1L KO) (Fig. 8 A). Lastly, apoptosis was also rescued upon reintroduction of WT ISG15 but not with the introduction of Luc in the ISG15 KO cell line (Fig. 8 B). Additionally, when we treated our cells with a pan-caspase inhibitor (Z-VAD) together with 1,000 IU/ml of IFNα2b, we significantly ameliorated cell death of the ISG15 KO fibroblasts (Fig. 8 C).

Next, we used a live-cell imager (BioSpa) to image the cells every 12 h following IFNα2b treatment (Fig. 8 D). Upon accurate cell segmentation through CellPose, we analyzed the proliferation rate of A549 ISG15 KO cells compared with WT. It was evident that in the absence of IFNα2b, the ISG15 KO cells proliferated at a similar rate than nontreated WT, but once IFNα2b was introduced, the proliferation rate dropped to about half (Fig. 8 D).

Lastly, we confirmed the presence of active caspase 3 in the patient’s samples through IHC with an antibody specific to cleaved caspase 3. The higher levels of caspase 3 in the epidermis that we recorded through ST were in agreement with the presence of active caspase 3 in the basal layer of epidermis in the biopsies as indicated in brown in Fig. 8 E. Moreover, lesion 1 and especially lesion 2 showed elevated levels of cleaved (active) caspase 3 in the dermis (Fig. 8 E).

Albeit the predictive value of the scratch assays for clinical defects in wound healing is relatively low, our results suggest that due to the enhanced antiproliferative and proapoptotic effects of IFN-I in ISG15-deficient lesions, IFN-I prevents the migration and sealing of the wound and thus maintains an active wound. As such, our working model based on the ST and our in vitro assays suggests that activation of macrophages at the site of injury causes TGFβ release that acts in a paracrine fashion, inducing EMT and fibroblast activation. Activated myofibroblasts further secrete TGFβ, allowing them to maintain their myofibroblast phenotype and induce EMT in the neighboring epidermal keratinocytes.

In conclusion, we were able to recapitulate in our in vitro system the human skin phenotype we observed in ST. We documented high apoptotic cell death levels that were triggered by IFNα in ISG15-deficient cells and showed that this phenotype was dependent on free ISG15, and independent of ISGylation.

## Discussion

ISG15 deficiency is one of several monogenic diseases encompassing type I interferonopathies (1, 2). Whereas most type I interferonopathies share certain clinical features such as systemic IFN-I levels and basal ganglion calcifications, some other features can vary depending on the genotype (1, 20, 21, 22). Typically, ISG15-deficient patients’ skin lesions differ from the lesions seen in other interferonopathies that entail excessive IFN-I production, in that they are not chilblain-like. Additionally, a small number of ISG15-deficient patients presented with interstitial lung disease. Previous work supports the importance of ISG15 in skin architecture and integrity, which could be relevant to the pathophysiology of these patients (25). However, the exact pathological mechanisms behind these symptoms are still poorly understood (31). In this study, we describe a novel disease-causing ISG15 mutation and explore potential mechanisms underlying tissue damage and fibrosis associated with ISG15 deficiency.

Even though we were able to detect *ISG15* mRNA in RNA extracted from our patient’s whole blood, our functional assays revealed the loss of production of this protein, as indicated by the absence of free ISG15 and ISGylation in vitro. Moreover, our patient exhibited an elevated ISG score in the blood. Lung biopsy slides from this individual (or her brother who harbors the same biallelic ISG15 mutation) were unavailable, so we instead assessed skin biopsies from three lesions of a different ISG15-deficient patient, compared with nonlesional skin from a healthy control (23). H&E staining revealed shared skin layers between the patient’s biopsies and the healthy control. However, in two of the patient’s skin biopsies, we detected regions of abnormal cell death (nonspecific tissue damage and sclerosis). To take a deeper look into the complex skin pathophysiology of ISG15 deficiency, we performed ST on the slides of the patient’s biopsies, as well as the control.

We recorded elevated mRNA levels of apoptotic markers (*CASP3*, *CASP8*, *BAX*, *BAK1*, and *CYCS*) in the patient as compared to the control samples, with a more pronounced difference in the dermal region of the skin. Since one of the clinical features of ISG15 deficiency, and type I interferonopathies in general, is elevated systemic IFNα, we assessed the ISG score of this patient by looking at the levels of several different ISGs, such as *IFIT1*, *USP18*, *MX1*, *IFI27*, *IFI44L*, among others, all of which were highly elevated and covered around 60–90% of the patient’s skin biopsies but with no significant levels in the control. The enhanced apoptotic markers were more prominent in the inflamed areas of the biopsies, which could indicate that IFN-I–mediated inflammation is indeed, at least in part responsible for the cell death in ISG15 deficiency. In addition, the persistence and relative amount of local IFN-I could also contribute to the observed phenotypes. Nonetheless, we were able to validate in vitro the relative cytotoxicity of IFN-I in ISG15 deficiency. We recapitulated the enhanced activation of apoptosis, via cleaved caspase 3, in the ISG15 KO cells, as well as their impaired migratory capacity to repopulate an artificial wound in an ISGylation-independent fashion, without noticing any substantial contribution of necroptosis or autophagy. However, while the failure of necrostatin-1 in rescuing cell death is suggestive of the absence of necroptosis and RIPK1-dependent PANoptosis, this may be different in different cell types and will need further assessment.

As these cell- and tissue-intrinsic factors are important, we then sought to evaluate the relative influence of immune cells. We observed the elevated numbers of M1 macrophage markers in the CD68^+^ spots in combination with the higher percentage of CD68^+^ spots that express cytokines secreted by both M1 and M2 macrophages. These could indicate that ISG15-deficient patients exhibit enhanced and chronic activation of macrophages at the site of tissue damage, which prolongs the time needed for wound healing and contributes to fibrosis. Of specific interest was the elevated production of TGFβ from the myeloid (CD68^+^) compartment, as well as from the epidermis of the patient, with no substantial TGFβ levels in the control. Our in vitro experiments further validated our hypothesis, as we were able to convert ISG15 KO lung epithelial cells into myofibroblasts within 3 days of IFNα/TGFβ cotreatment, unlike the control cells. Lastly, prolonged treatment of fibroblasts with IFNα/TGFβ also led to myofibroblast activation in the ISG15 KO cells but not in the WT counterpart. This led to our working model where, due to the chronic wound—which can be maintained in ISG15 deficiency due to the exacerbated IFN-I effects that prevent the migration and proliferation of those cells—and improper macrophage activation, TGFβ is produced and secreted by the macrophages, triggering EMT of the proximal epidermis, which once transdifferentiated into myofibroblasts further enhance the production of TGFβ, thus propagating the EMT of neighboring areas and intensifying fibrosis.

In conclusion, ISG15 deficiency is a complex monogenic syndrome that poises individuals to a proapoptotic and profibrotic state that can lead to lung and skin damage with scarring and impaired function. This can be a life-threatening condition, as severe lung fibrosis can lead to irreversible loss of lung functionality with lung transplantation as the only solution. In addition, chronic wounds predispose patients to infections, and skin fibrosis leads to loss of hair follicles, sweat glands, and other skin components crucial for homeostasis. Further research is required to elucidate how IFN-I affects each of the cell types independently and in concert, and whether these processes could be exacerbated by commensal or typical infection of childhood and adulthood.

## Materials and methods

### qPCR

Total RNA was isolated either from cells in culture with RNeasy spin columns (74104; QIAGEN) or from whole blood with the PAXgene Blood RNA kit (762164). High-Capacity RT Kit (4368814; Applied Biosystems) was used to synthesize cDNA, and the expression levels of various genes relative to either *18S* (4318839) or *GAPDH* (4453320) RNA housekeeping genes were analyzed with the TaqMan Master Mix II with UNG (4440038; Thermo Fisher Scientific) on a LightCycler 480. The relative expression levels of those genes were calculated by the ΔΔCT method in comparison with the mean value of the untreated healthy control. The probes used were the following: *TGFB1* Hs07289533_m1, *ISG15* Hs01921425_s1, *IFIT1* Hs03027069_s1, *IFI27* Hs01086373_g1, *SIGLEC1* Hs00988063_m1, *MX1* Hs00895608_m1, *FGF-2* Hs00266645_m1, *ITGB6* Hs00168458_m1, *PDGFA* Hs00234994_m1.

### 3′-RACE PCR

Total RNA was extracted from the patient’s whole blood as described above, and 3′-RACE (18373019; Invitrogen) was used for the amplification of nucleic acid sequences from mRNA. We used the 3′-RACE universal amplification primer supplied in the kit, which was complementary to the adaptor primer and an ISG15-specific primer.

### Plasmids and transfection

HEK293T cells were transfected with Lipofectamine 2000 (Invitrogen). The variants were introduced with the QuikChange II XL site-directed mutagenesis kit (200522; Agilent Technologies), using the pDONOR221-ISG15 vector. The variants were then transferred into a pTRIP expression vector with the Gateway LR Clonase II enzyme mix (11791100; Thermo Fisher Scientific).

### Western blot

Cells were seeded at a cell density of 2 × 10^5^ cells per well in a 6-well plate. Following the appropriate treatments for each experiment, cells were lysed in radioimmunoprecipitation assay buffer (89900; Thermo Fisher Scientific) with Dithiothreitol (DTT) and protease/phosphatase inhibitor cocktail (#5872; Cell Signaling) for at least 20 min on ice. Samples were centrifuged to remove insoluble complexes and then boiled with NuPAGE sample buffer (NP0007; Thermo Fisher Scientific) containing 50 mM DTT. The samples were then subjected to western blotting and semi-dry transfer. Membranes were blocked in 5% milk, and following an overnight incubation with primary antibodies, the membranes were washed three times with wash buffer (PBS with 0.1% Tween-20 and 10X TBS), incubated for 1 h with HRP-conjugated secondary antibodies, washed three times (5–10 min each wash), and visualized on film.

The primary antibodies used were the following: anti-ISG15 (F-9) (sc-166755; Santa Cruz), anti-LC3B (#2775; Cell Signaling), anti-GAPDH (D16H11) (#5174; Cell Signaling).

Fig. 1 H is a representative of at least three replicate experiments. Fig. 7 F is a representative of a replicate experiment.

### Spatial transcriptomics

#### Sample processing

Three patient-derived and one healthy control 10-μm-thick FFPE sections were submitted to Mount Sinai’s Center for Advanced Genomic Technology Core and were mounted onto 10× Visium-based ST array slides for spatial gene expression analysis. FFPE tissue section–compatible 10× library protocol was used for processing the samples, and RNA expression/assay was captured. Moreover, the ST dataset of two additional healthy skin biopsies, HV2.S1.R1 and HV3.S1, referred to as control 2 and control 3, respectively, in this manuscript, was obtained from the GSE202011 repository (32).

#### Data analysis

A graphical representation of the overall data analysis showing the general pipeline and figures generated at each point can be found in Fig. S2 A. Sequencing output and the histology images were processed using Space Ranger software (10x Genomics). The mkfastq function from the Space Ranger pipeline was used for sample demultiplexing, and spatial barcodes and reads were converted into FASTQ format. The Space Ranger count function was used for read alignment and calculating gene counts on the basis of the human reference genome (GRCh38) and then aligning microscopic slide images and transcriptomes to generate spot barcode/Unique Molecular Identifier (UMI) counts and feature spot matrices.

The output from Space Ranger was then processed downstream in an R programming environment, and the Seurat package was used for primary analysis. Standard quality control steps were performed, including generating *R*^2^ plots for all ST samples to evaluate the concordance between UMI counts and the number of features detected on a per-spot basis. Spots with poor readout (below [200 genes/features detected per spot]) were removed prior to downstream analysis as part of the quality control step.

The complete dataset consisted of three healthy/control samples and three ISG15-deficient patient samples. Each sample was normalized independently using the SCTransform function in the Seurat package with default parameters as described in the vignette. Following that, the SelectIntegerationFeature function was used to find features to be used for batch correction downstream from all the samples, and these features were selected as Variable Features for PCA. After running PCA, batch correction was performed using Harmony algorithm by running the RunHarmony Seurat function using default parameters, Single Cell Transform (SCT) counts as assay and group variable set to orig.ident (i.e., the sample ID) in metadata to remove sample-specific batch effect. The top 20 dimensions from Harmony embeddings were used for the UMAP-based dimensionality reduction and FindNeighbors function. To determine the best resolution setting for FindClusters, we constructed a cluster tree using the clustree package (82), showing different clusters’ clustering resolutions from 0 to 1 (Fig. S2 B). Based on the clustree visualization, we found optimal resolution as 0.7 and then manually annotated clusters using cluster-specific marker genes after running the FindAllMarkers function using default parameters with test set to Wilcoxon’s rank sum statistical test, minimum cell percentage set to 25%, and log2 fold change cutoff set to 0.25. The clusters were also visualized on ST slide by running the SpatialDimPlot function (Fig. 3 A) and also using the DimPlot function to generate a UMAP plot showing different samples and clusters (Fig. S2 A).

Gene expression showing ISGs was visualized using the DoHeatmap function to generate heatmaps (Fig. S3 C). The SpatialFeaturePlot function was also used to visualize myofibroblast markers for each biopsy (Fig. 5 A and Fig. S5 A), as well as the DoHeatmap function for heatmaps (Fig. 5 B).

The anchor-based single-cell deconvolution method from the Seurat package was used to generate prediction scores for all annotated cell types in the reference single-cell dataset (GEO: GSE150672) (83). These cell-type deconvolution prediction scores were then visualized using the SpatialFeaturePlot function (Fig. S2 D and Fig. 4 A).

Multimodal integration analysis (MIA) was used to assess the overlap of marker genes between each ST cluster and scRNA-seq cluster (64). MIA generated an enrichment score by using the top 300 upregulated marker genes (Fig. S2 C). We set our adjusted P value to be ≤0.05 and log fold change >0.25 for all marker genes used in MIA.

We run the PrepSCTFindMarkers function before the FindAllMarkers function.

Pathway analysis was performed using Enrichr, and results were filtered by p-adjusted value <0.05 (Fig. 5 D and Fig. 5 A). P-adjusted values were computed using the Benjamini–Hochberg method of correction for multiple hypothesis testing.

MultiBarHeatmaps (Fig. 5 B and Fig. S3 C) were generated using the DoMultiBarHeatmap function (https://github.com/elliefewings/DoMultiBarHeatmap).

The ST dataset was deposited in GEO under the accession number GSE283022.

### IHC

Formalin-fixed paraffin-embedded tissues were cut into 3-μm sections and stained by the Biorepository and Pathology Core (Icahn School of Medicine at Mount Sinai, New York, NY, USA).

#### Single IHC staining

The presence of caspase 3 was probed with anti-caspase 3 antibody (Cat. PP229AA; Biocare Medical). Briefly, IHC staining was performed using VENTANA DISCOVERY ULTRA automated slide staining instrument (Roche). Single semi-automatic staining was performed using the selected primary antibody and the Discovery OmniMap anti-host-HRP (Roche) as secondary antibody. The signal was obtained using Discovery ChromoMap DAB RUO (760-2513) (depicted in brown signal). Tissues were counterstained with hematoxylin to visualize the nuclei.

#### Multiplex IHC staining

For sequential chromogenic multiplex IHC, the following markers were evaluated: CD16(Sp175) (Cat. 760-4863), CD163(MRQ-26) (Cat. 760-4437), and actin, smooth muscle (1A4) (prediluted from Roche) using VENTANA DISCOVERY ULTRA automated slide staining instrument (Roche). Each primary marker was followed by the corresponding secondary antibody: DISCOVERY OmniMap anti-Rb HRP (RUO) (Cat. 760-4311), DISCOVERY anti-rabbit HQ (Cat. 760-4815), DISCOVERY anti-mouse HQ (Cat. 760-4814), and the signal was developed with a different color, respectively: DISCOVERY ChromoMap DAB Kit (RUO) (Cat. 760-159; brown), DISCOVERY Purple Kit (RUO) (Cat. 760-229; purple), and DISCOVERY Green HRP Kit (Cat. 760-271; green). Importantly, after each sequential staining, slides went through a step of inhibition, heat denaturation, and neutralization.

Tissues were counterstained with hematoxylin to visualize the nuclei.

Whole-tissue sections on the slide were converted into high-resolution digital data using a NanoZoomer S210 Digital slide scanner (Hamamatsu).

The HALO image analysis platform and HALO AI (HALO Artificial Inteligence) were used for quantitative tissue analysis (Indica Labs, Inc.), using AI for nucleus segmentation. Multiplex IHC module and color deconvolution were used to separate chromogenic stains together with nucleus segmentation to set up the system for quantitative analysis and determination of unsupervised pixelwise H-score values.

Representative multiplex staining for CD16, CD163, and ACTA2 is shown in Fig. 4 E, while the corresponding individual stains for each marker are presented in Fig. S3 E. For some samples (control 1, control 3, lesion 1, and lesion 2), the regions shown in Fig. 4 E overlap with those displayed in Fig. S3 E. For control 2 and lesion 3, different representative regions of the biopsies are shown; however, these images convey the same findings.

### EMT

A549 cells were seeded in a 12-well plate at a density of 1 × 10^4^ cells per well. The following day, cells were treated with IFN-α2b (100 or 1,000 IU ml^−1^, Merck IntronA), TGFβ (10 ng ml^−1^, 75362; Cell Signaling), or both IFNα and TGFβ together. Images on an EVOS microscope at ×10 magnification were taken daily for 5 days. CellPose was then used to analyze EMT.

### Flow cytometry

hTERT fibroblasts were seeded in 12-well plates at a density of 5 × 10^4^ cells per well. The following day, they were incubated with 0, 100, or 1,000 IU ml^−1^ IFN-α2b (Merck IntronA) in normal growth medium for 48 or 72 h, prior to fixation and permeabilization with BD Cytofix/Cytoperm Kit (554714; BD Biosciences). Samples were then stained with cleaved caspase 3 antibody conjugated with FITC (×12 dilution, 5168654X; BD Pharmingen) in wash buffer provided by the BD Biosciences Cytofix/Cytoperm kit for 40 min at 4C and protected from light.

Alternatively, cells were stained for Annexin V/PI as indicated by Invitrogen (V13245).

### Deep Blue assay

Cell death assay cells were plated at 80–90% confluency in a 96-well plate the day before. Relevant samples were pretreated with 10 mM of the RIPK1 inhibitor, necrostatin-1 (S8037; Selleck Chemicals) for 30 min before an 48-h stimulation with 1,000 IU ml^−1^ IFN-α2b (Merck IntronA). Viability was measured with Deep Blue Cell Viability Kit (424702; BioLegend) on a Synergy H1 Hybrid Reader (BioTek).

### Scratch assay

Fibroblasts were seeded at a cell density of 1 × 10^4^ cells per well in a 96-well plate, left at room temperature for 30 min to allow even distribution of cells, and then incubated overnight at 37°C. Cells were treated with various amounts of IFNα (Merck IntronA) for 6 h prior to the scratch assay. The scratch assay was performed as indicated by Sartorius and imaged every hour with Incucyte plate imager. There were at least six replicated for each condition for each experiment.

Fig. 6, A and D, is representative of at least three replicate experiments.

### Statistical analysis

Statistical analysis and P value calculation were performed with GraphPad Prism v.10.5.0. Error bars indicate the standard deviation. For all cell-based assays, P values were calculated with a two-tailed *t* test. *P < 0.05; **P < 0.01; ***P < 10^−3^.

### Online supplemental material

Fig. S1 shows ST deconvolution of cell types and cluster validation; Fig. S2 shows additional evidence of IFN-I–mediated inflammation at the ST level; Fig. S3 shows the markers used to validate the presence of myeloid cells in ST and IHC; Fig. S4 shows additional myofibroblast markers in ST; Fig. S5 shows additional data from pathway enrichment analysis.

## Supplementary Material

SourceData F1 is the source file for Fig. 1.

SourceData F7 is the source file for Fig. 7.

## Data Availability

The ST data were deposited in GEO (GSE283022). A publicly available single-cell dataset was used for cell-type annotation in the ST data analysis (GEO repository, GSE150672) (83). All other data are either included in the manuscript or available upon reasonable request.
