# Supplementary material for: Human ISG15 deficiency unveils impaired healing of ulcerations via type I interferon–mediated fibrosis
Source: J Hum Immun. 2026 Feb 24;2(3):e20250011. doi: 10.70962/jhi.20250011 (PMC12931375; doi:10.70962/jhi.20250011)
Supplement: SourceData F1 — is the source file for Fig. 1. [file jhi_20250011_sourcedataf1.pdf]

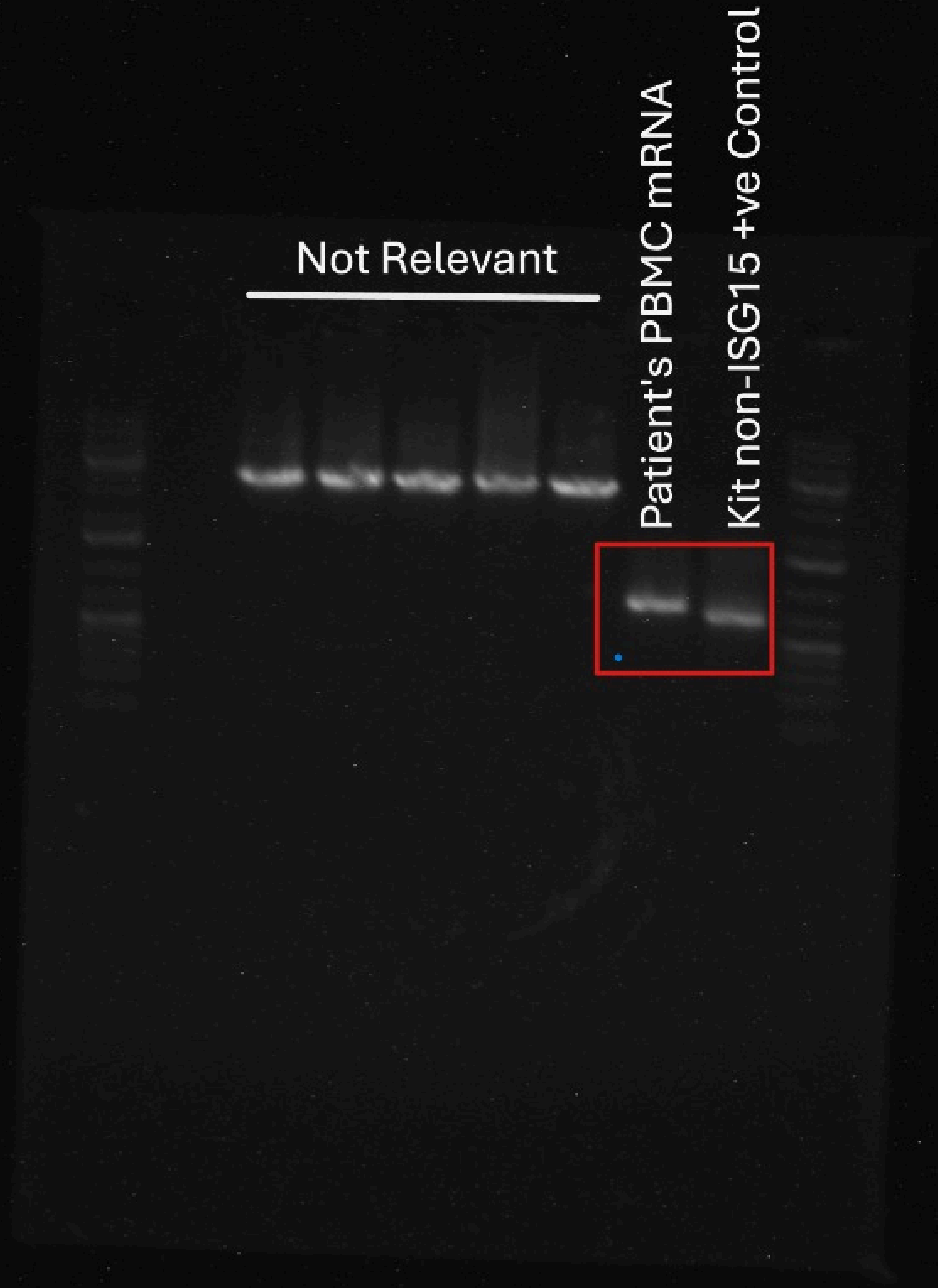
Not Relevant
Patient's PBMC mRNA
Kit non-ISG15 +ve Control

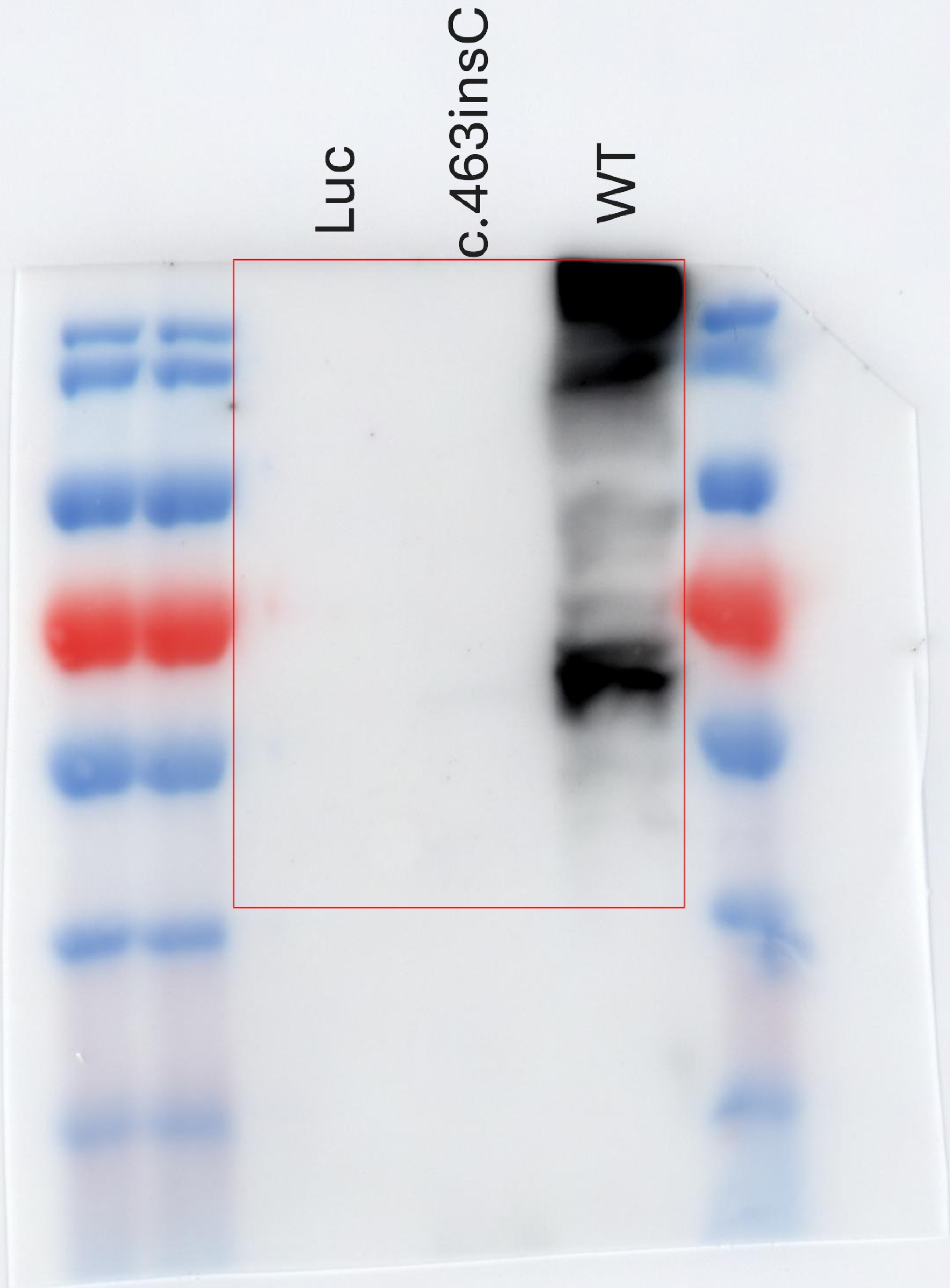

ISGylation

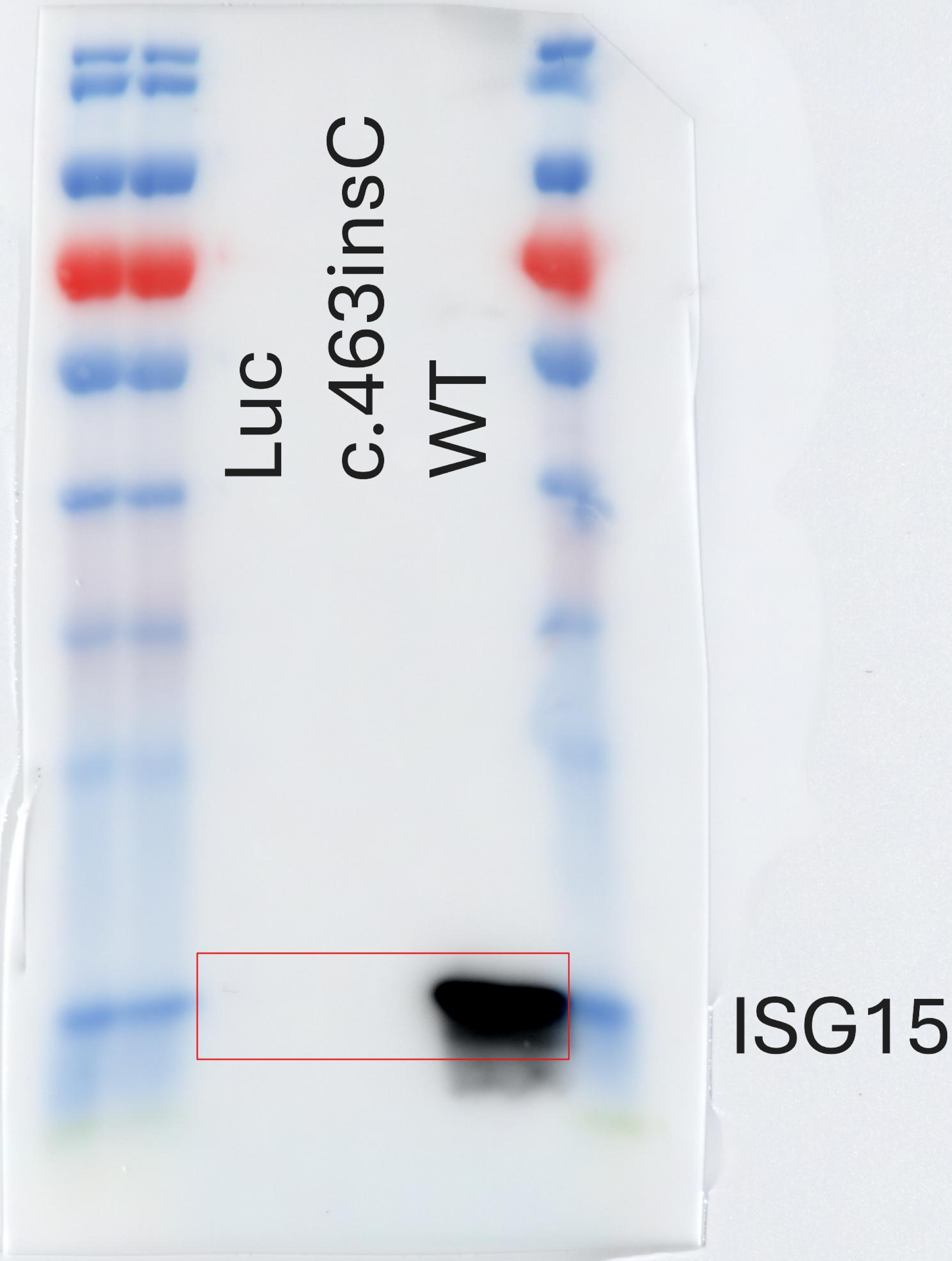
Luc
c.463insC
WT
ISG15

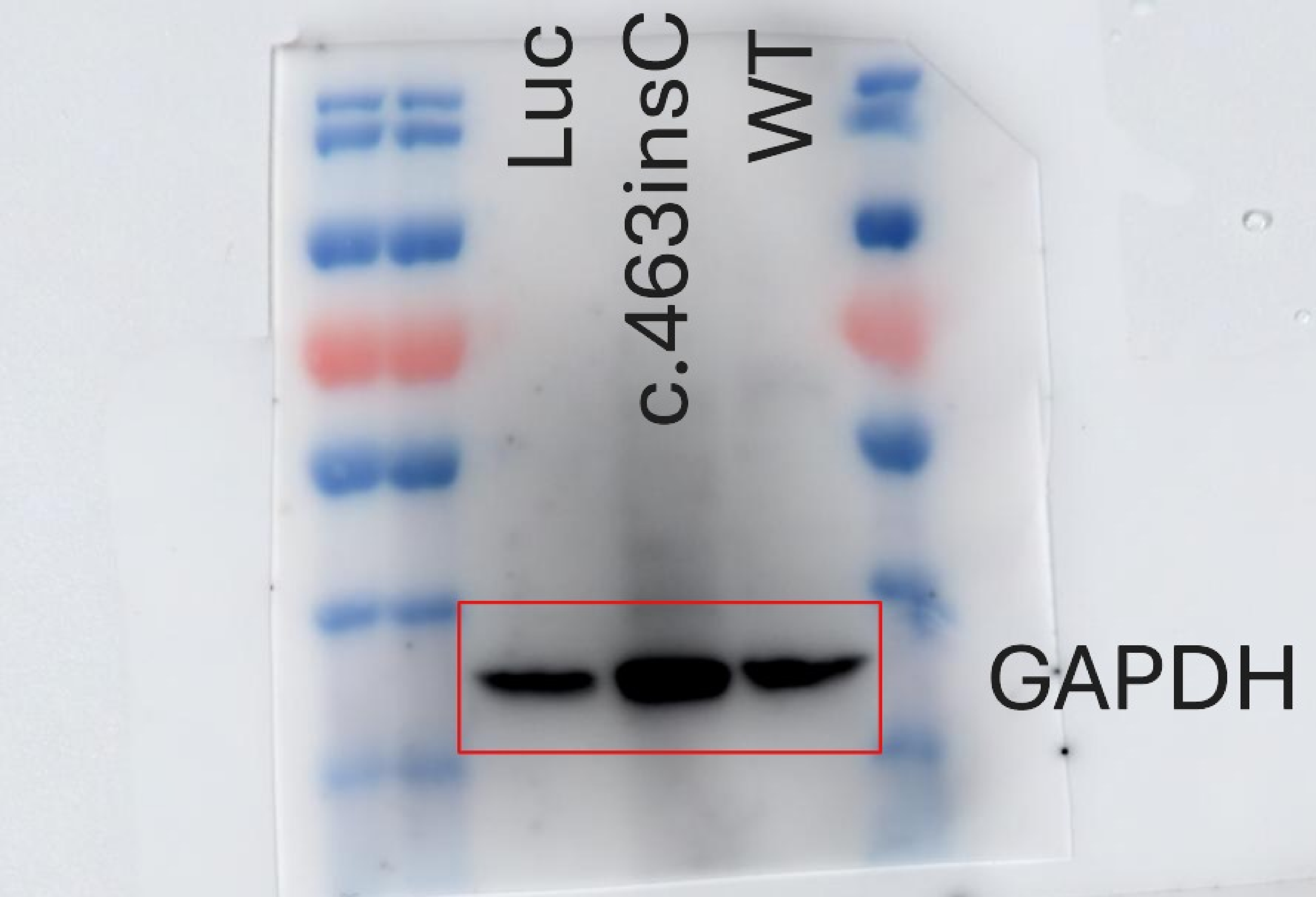
Luc
c.463insC
WT
GAPDH
