# Supplementary figures and images for: Human ISG15 deficiency unveils impaired healing of ulcerations via type I interferon–mediated fibrosis
Source: J Hum Immun. 2026 Feb 24;2(3):e20250011. doi: 10.70962/jhi.20250011 (PMC12931375; doi:10.70962/jhi.20250011)

ISG15

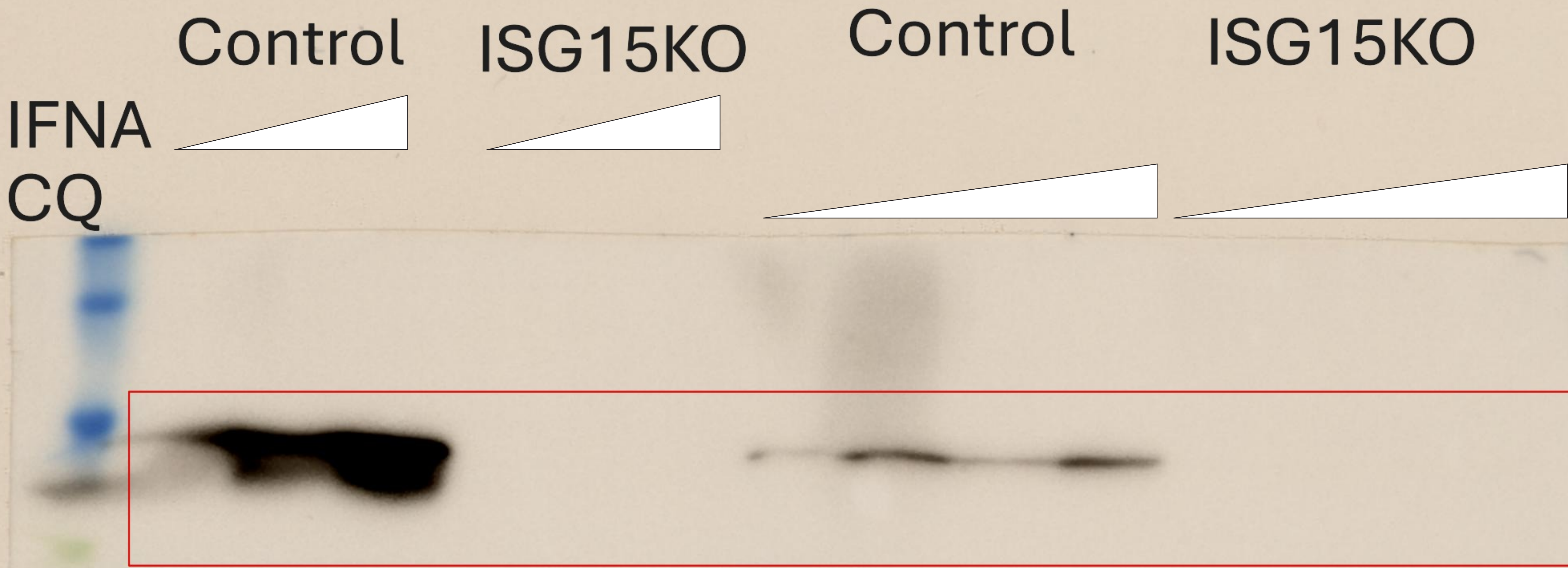

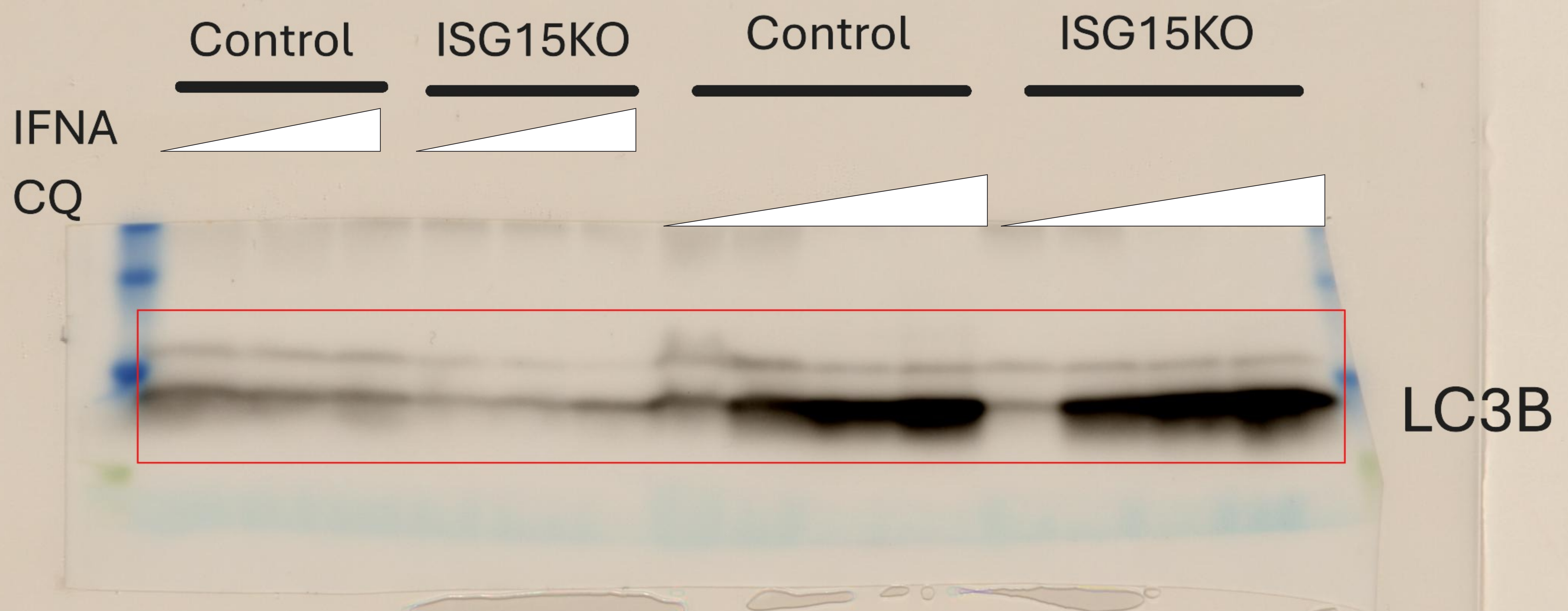

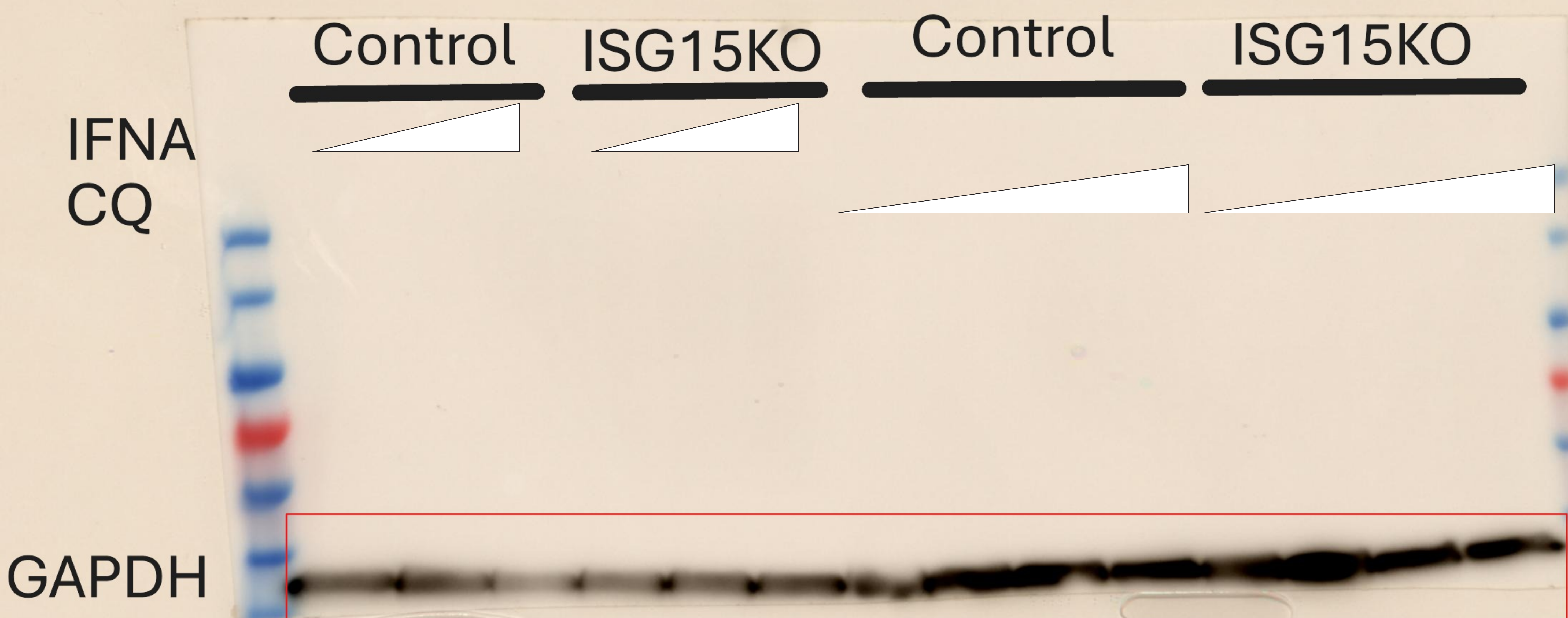

Supplement: SourceData F7 — is the source file for Fig. 7. [file jhi_20250011_sourcedataf7.pdf]
